# Targeting the TRIM25–AGO2–miR-148b-5p–ABCC1 axis overcomes chemoresistance in non-small cell lung cancer

**DOI:** 10.1038/s41419-026-08802-1

**Published:** 2026-04-30

**Authors:** Zihan Zhou, Ran Chen, Lian Li, Runhui Lu, Hongyan Li, Junya Li, Yingting Cao, Yixin Zhang, Xiangling Jiang, Anan Xu, Yun Yi, Yanli Wang, Jian Huang, Xiaojing Zhao, Chunling Du, Jianxiu Yu

**Affiliations:** 1 Department of Biochemistry and Molecular Cell Biology & Department of Thoracic Surgery Ren Ji Hospital, Shanghai Jiao Tong University School of Medicine, Shanghai, China; 2 Department of Respiratory and Critical Care Medicine, QingPu Branch of Zhongshan Hospital Affiliated to Fudan University, Shanghai, China; 3 Center of Biobank, The Second Affiliated Hospital of Nanchang University, Nanchang, China; 4 Guangzhou Glowsi Biotechnology Co. Ltd, Guangzhou, China

**Keywords:** Non-small-cell lung cancer, Ubiquitylation

## Abstract

Despite significant advances in the treatment of non-small cell lung cancer (NSCLC), acquired resistance remains a major obstacle in advanced stages. Elucidating the molecular mechanisms underlying resistance is crucial for improving clinical outcomes, overcoming therapeutic limitations, and developing effective combination strategies. Here, we identify TRIM25 as a key driver of tumor progression and chemoresistance by promoting the destabilization of AGO2. Mechanistically, TRIM25 directly binds to AGO2 and induces its polyubiquitination, triggering proteasomal degradation. This process downregulates miR-148b-5p, a tumor-suppressive miRNA that post-transcriptionally represses ABCC1 to counteract chemoresistance. Knockdown of TRIM25 restores AGO2 stability by attenuating ubiquitination, thereby reinstating miR-148b-5p-mediated suppression of ABCC1. Functionally, the TRIM25–AGO2–miR-148b-5p–ABCC1 axis inhibits tumor growth and re-sensitizes NSCLC cells to chemotherapy. Notably, therapeutic delivery of miR-148b-5p mimics robustly suppresses NSCLC progression and overcomes chemoresistance in cell lines, xenografts, and patient-derived xenograft (PDX) models, highlighting its translational potential. Our study unveils a previously unrecognized regulatory axis governing chemoresistance in NSCLC, providing both mechanistic insights and novel therapeutic avenues to combat treatment resistance.

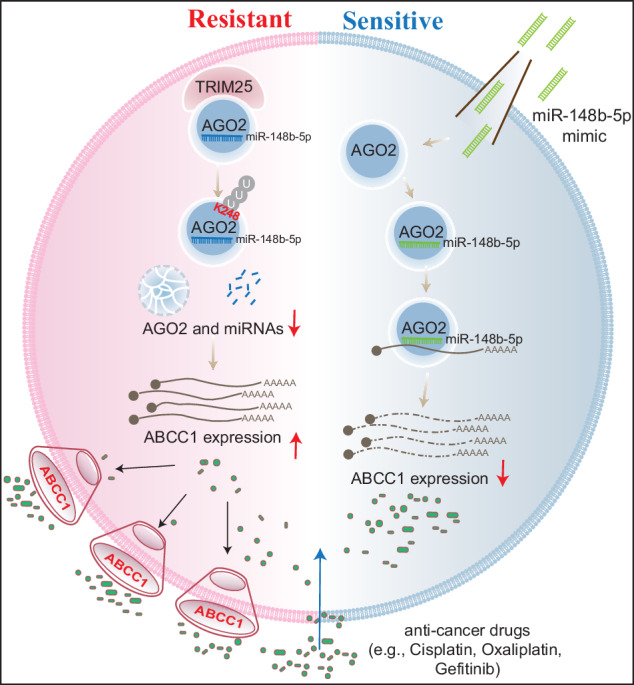

## Introduction

Lung cancer is the leading cause of cancer-related deaths worldwide, with the highest mortality rates among both men and women [1, 2]. Histologically, it is classified into two major subtypes: small-cell lung cancer (SCLC) and non-small-cell lung cancer (NSCLC). NSCLC accounts for approximately 85% of all lung cancers [3, 4], with adenocarcinoma (50%) and squamous cell carcinoma (20–30%) being the most common histological types [5]. For early-stage NSCLC, surgical resection remains the standard treatment, while chemotherapy is widely used for advanced NSCLC [4, 6]. Although chemotherapy drugs have improved survival duration and reduced adverse events, resistance to single-agent therapies remains a major challenge [7]. Drug combinations provide a promising strategy to overcome resistance [8, 9], highlighting the need to elucidate the molecular mechanisms underlying therapy resistance and develop more effective combination therapies for improved cancer treatment.

One of the primary mechanisms underlying drug resistance and chemotherapeutic failure in cancer treatment is the ATP-binding cassette (ABC) transporter-mediated efflux of chemotherapeutic agents, which reduces their intracellular accumulation [10, 11]. To date, 51 human ABC transporters have been identified and characterized, classified into seven distinct subfamilies (ABCA through ABCG) based on their genetic and structural organization. The canonical ABC transporter structure features a four-domain architecture: two cytoplasmic nucleotide-binding domains (NBDs) that mediate ATP binding and hydrolysis, and two transmembrane domains (TMDs) responsible for substrate recognition and transport [11–13]. Among these, ABCB1 (P-glycoprotein, P-gp), ABCC1 (multidrug resistance-associated protein 1, MRP1), and ABCG2 (breast cancer resistance protein, BCRP) are particularly noteworthy for their frequent overexpression in multidrug-resistant (MDR) cancers. These transporters, which demonstrate broad substrate specificity and play a crucial role in conferring resistance to diverse anticancer agents [11], have been extensively studied. Given their clinical significance, developing non-toxic ABC transporter inhibitors that can be co-administered with conventional chemotherapeutics represents a promising strategy to overcome MDR and enhance treatment efficacy.

**Fig. 1:**
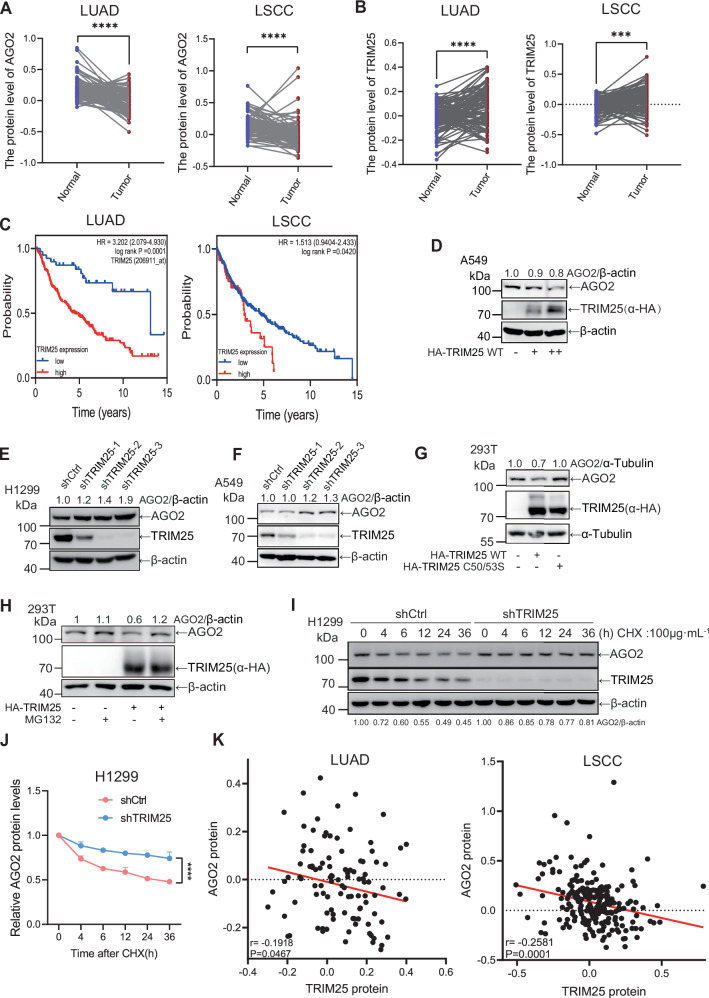
TRIM25 negatively regulates AGO2 protein levels in cancer cells. **A** Protein levels of AGO2 were decreased in LUAD and LSCC. Statistical analysis was performed using a two-tailed unpaired *t*-test, ^****^P < 0.0001. **B** Protein levels of TRIM25 were increased in LUAD and LSCC. Statistical analysis was performed using a two-tailed unpaired *t*-test, ^****^P < 0.0001. **C** Kaplan–Meier survival curves comparing overall survival between patients with high (red) and low (blue) TRIM25 expression levels in (left) LUAD (PrognoScan microarray data) and (right) LSCC (OncoLnc RNA-seq data). TRIM25 (206911_at) refers to the Affymetrix probe set ID 206911_at corresponding to the TRIM25 gene in the LUAD microarray dataset. **D** Western blotting analysis for endogenous AGO2 in A549 cells transfected with HA-TRIM25. **E**, **F** Western blotting analysis for endogenous AGO2, TRIM25 in H1299 (**E**) and A549 (**F**) stable cells with TRIM25 knockdown. **G** Western blotting analysis for endogenous AGO2 in 293T cells transfected with HA-TRIM25^WT^ or HA-TRIM25^C50/53S^. **H** HA-TRIM25 was transfected into 293T cells, followed by the treatment by adding 20 μM MG132 for 4 h. **I**, **J** The half-life of endogenous AGO2 protein was determined in TRIM25-knockdown H1299 cells with treatment of CHX (100 μg·mL^−1^) for the indicated time points (**I**), and quantified and normalized to β-actin with Image J (**J**). MG132 pretreatment prior to CHX was used to normalize protein levels. Data were presented as means ± SD, *n* = 3. Statistical analysis was performed using one-way ANOVA. ^****^P < 0.0001. Band intensities were quantified by ImageJ software. **K** Linear regression analysis of the patients with LUAD and LSCC showed a negative correlation between AGO2 and TRIM25 proteins, pearson coefficient, and *P* values were indicated.

Our most recent study demonstrated that Tank-binding kinase 1 (TBK1) phosphorylates AGO2 at serine 417 (pS417-AGO2) following Gefitinib treatment, promoting NSCLC progression and conferring Gefitinib resistance through enhanced formation and activity of oncogenic microRNA-induced silencing complexes (miRISCs) [14]. However, the precise mechanisms underlying AGO2-mediated drug resistance in cancer cells remain incompletely understood. Analysis of AGO2 protein expression patterns in the Clinical Proteomic Tumor Analysis Consortium (CPTAC) database showed significant downregulation in lung adenocarcinoma (LUAD), lung squamous cell carcinoma (LSCC), and breast cancer (BRCA) specimens (Figs. 1A and S1A). Despite these findings, the biological implications and molecular mechanisms driving AGO2 downregulation in cancer pathogenesis require further investigation.

The tripartite motif-containing (TRIM) protein family plays crucial roles in diverse biological processes, including cell proliferation, differentiation, apoptosis, tumorigenesis, and innate immunity, largely through regulating protein quality control [15, 16]. TRIM25, a structurally conserved family member, contains four characteristic domains: an N-terminal RING finger domain, two B-box domains, a coiled-coil domain (CCD), and a C-terminal PRYSPRY domain [17]. Although TRIM25 was initially characterized for its role in antiviral immunity *via* mediating Lys63-linked ubiquitination of RIG-I [18, 19], emerging evidence demonstrates its critical involvement in cancer progression [20, 21].

This study demonstrates that TRIM25 directly interacts with AGO2 and promotes its polyubiquitination, thereby destabilizing AGO2 *via* the ubiquitin-proteasome pathway. Clinically, elevated TRIM25 expression correlates with poor patient prognosis and confers drug resistance by modulating the AGO2–miR-148b-5p–ABCC1 axis. These findings underscore the therapeutic potential of targeting this regulatory network for improved cancer treatment strategies.

## Results

### TRIM25 negatively regulates AGO2 protein levels in cancer cells

To identify potential regulators of AGO2 protein stability, we overexpressed Flag-tagged AGO2 in HEK293T cells and performed anti-Flag immunoprecipitation coupled with high-sensitivity mass spectrometry (MS). Our proteomic analysis revealed several E3 ubiquitin ligases that co-purified with AGO2 (Fig. S1B), implicating their possible roles in modulating AGO2 stability. Notably, TRIM25 showed elevated expression in tumor tissues from LUAD, LSCC, and BRCA patients, as indicated by CPTAC database analysis (Figs. 1B and S1C). Consistent with a possible oncogenic role, Kaplan–Meier survival analysis linked high TRIM25 expression to poorer patient prognosis (Figs. 1C and S1D). Given the well-established role of TRIM family proteins in mediating substrate degradation *via* the ubiquitin-proteasome pathway [22–25], we hypothesized that TRIM25 might similarly target AGO2 for proteasomal degradation. Supporting this hypothesis, increasing TRIM25 expression in A549 cells reduced AGO2 levels in a dose-dependent manner (Fig. 1D). Conversely, stable TRIM25 knockdown using shRNAs in H1299, A549, and DU145 cells increased AGO2 protein accumulation (Figs. 1E, F and S1E), further confirming TRIM25’s role in regulating AGO2 stability.

To assess whether TRIM25 functions as an E3 ubiquitin ligase in AGO2 regulation, we generated an enzymatically inactive mutant (C50S/C53S, TRIM25^C50/53S^) based on previous studies [25, 26]. Unlike wild-type TRIM25 (TRIM25^WT^), which reduced AGO2 levels in HEK293T cells, TRIM25^C50/53S^ had no effect (Fig. 1G), demonstrating that TRIM25-mediated AGO2 degradation depends on its E3 ligase activity. Notably, the proteasome inhibitor MG132, but not the lysosome inhibitor chloroquine (Fig. S1F), reversed TRIM25-induced AGO2 reduction (Fig. 1H), confirming proteasome-dependent degradation. To further define TRIM25’s role in AGO2 stability, we performed cycloheximide (CHX) chase assays. TRIM25 knockdown slowed AGO2 degradation in H1299 cells (Fig. 1I, J), while TRIM25 overexpression markedly shortened AGO2’s half-life (Fig. S1G, H). Together, these findings establish TRIM25 as a key destabilizer of AGO2 in cells.

To clinically validate these findings, we analyzed CPTAC data, which showed a significant inverse correlation between TRIM25 and AGO2 protein levels (Figs. 1K and S1I). This clinical observation corroborates our experimental data, firmly establishing TRIM25 as a negative regulator of AGO2 in cancer cells.

### TRIM25 directly binds AGO2

**Fig. 2:**
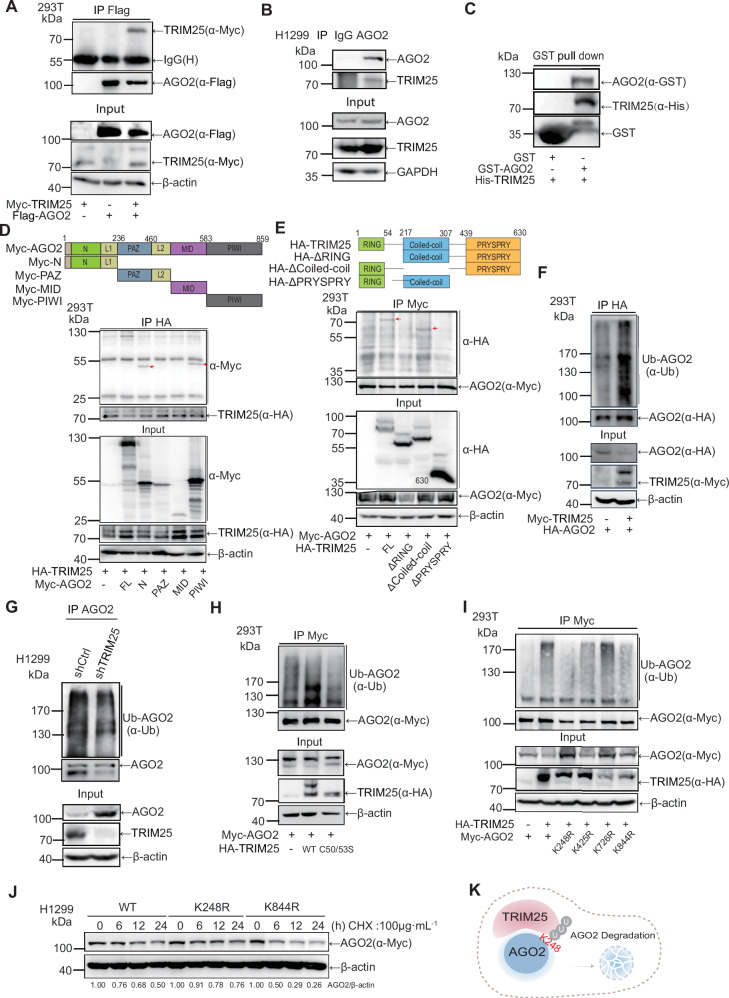
TRIM25 binds and ubiquitinates AGO2 at K248 to drive proteasomal degradation. **A** Flag-AGO2 was transfected alone or in combination with Myc-TRIM25 into 293T cells, followed by immunoprecipitation with the Flag antibody and immunoblotting analysis with Myc antibody. **B** Endogenous AGO2 was immunoprecipitated from H1299 cells and followed by immunoblotting with an antibody against TRIM25. **C** Purified GST-AGO2 was incubated with His-TRIM25 for the pull-down assay. **D** 293T cells transfected with HA-TRIM25 and different Myc-AGO2 truncated constructs were immunoprecipitated with anti-HA antibody, followed by western blotting analysis with anti-Myc antibody. **E** 293T cells transfected with Myc-AGO2 and different HA-TRIM25 truncated constructs were immunoprecipitated with anti-Myc antibody, followed by western blotting analysis with anti-HA antibody. **F** 293T cells were transfected with HA-AGO2 and Myc-TRIM25, followed by immunoprecipitation with the anti-HA antibody and probed with the anti-ubiquitin antibody. **G** Lysates from stable H1299-shTRIM25 cell lines were immunoprecipitated with anti-AGO2 antibody, followed by western blotting analysis with anti-ubiquitin antibody. **H** Lysates from 293T cells transfected with wild-type TRIM25 or mutant TRIM25^C50/53S^ were immunoprecipitated with anti-Myc antibody, followed by western blotting analysis with anti-ubiquitin antibody. **I** Immunoprecipitation was performed to detect the ubiquitination of AGO2 in 293T cells transfected with plasmids encoding HA-TRIM25 and Myc-AGO2 (wild-type or its mutants). **J** H1299 cells were transfected with Myc-AGO2 (WT or its mutants) for 24 h, followed by treated with CHX for the indicated time. AGO2 bands were quantified by ImageJ software. Band intensities were quantified by ImageJ software. Quantification of Western blot results and corresponding statistical analyses are presented in Fig. S2D. **K** Schematic diagram of TRIM25 regulating AGO2 degradation.

Having identified TRIM25 as a regulator of AGO2 stability, we employed multiple approaches to validate its physical interaction. Initial co-immunoprecipitation (Co-IP) experiments in HEK293T cells expressing Flag-AGO2 and Myc-TRIM25 confirmed this association (Fig. 2A). The interaction was further supported by Co-IP of HA-AGO2 with endogenous TRIM25 in 293T cells (Fig. S2A) and by Co-IP of endogenous proteins in H1299 cells (Fig. 2B). A direct binding event was demonstrated by GST pull-down assays using bacterially expressed GST-AGO2 and His-TRIM25 (Fig. 2C). Domain mapping revealed that AGO2 primarily interacts with TRIM25 through its N and PIWI domains (Fig. 2D), while TRIM25 binds AGO2 via its RING and PRY/SPRY domains (Fig. 2E). Collectively, these data demonstrate that TRIM25 directly interacts with AGO2.

### TRIM25 ubiquitinates AGO2 at K248 to trigger degradation

Given that TRIM25 directly interacts with and destabilizes AGO2, we next asked whether it ubiquitinates AGO2. To test this, we co-expressed Myc-TRIM25 with HA-AGO2 in 293T cells and observed enhanced AGO2 ubiquitination (Fig. 2F). This effect was dose-dependent, with higher TRIM25 expression leading to increased AGO2 ubiquitination (Fig. S2B). Conversely, TRIM25 knockdown decreased AGO2 ubiquitination (Fig. 2G). Importantly, the RING domain mutant TRIM25^C50/53S^ failed to promote AGO2 polyubiquitination (Fig. 2H), demonstrating that TRIM25’s E3 ligase activity is essential for this function.

To determine the ubiquitination linkage specificity of TRIM25 on AGO2, we employed His-tagged ubiquitin mutants (K48-only and K63-only) containing arginine substitutions at all lysines except K48 or K63. These experiments demonstrated that TRIM25 mediates both K48- and K63-linked polyubiquitination of AGO2 (Fig. S2C).

Using ubiquitination site prediction analysis (http://ubibrowser.ncpsb.org.cn), we generated four AGO2 lysine-to-arginine mutants (K248R, K425R, K726R, K844R). While the K248R mutation completely abolished TRIM25-dependent ubiquitination, K844R only partially reduced it (Fig. 2I). Notably, only K248R exhibited enhanced protein stability in CHX chase assays (Figs. 2J and S2D), identifying K248 as the key residue regulating AGO2 degradation. Structural analysis (PDB: 4W5O) confirmed that K248 is surface-exposed (Fig. S2E), supporting its role as the primary ubiquitination site. Together, these findings demonstrate that TRIM25 selectively targets accessible K248 to mediate AGO2 polyubiquitination and proteasomal degradation (Fig. 2K).

### PI3K-AKT signaling stabilizes AGO2 by phosphorylating TRIM25 at S158 to block ubiquitination

**Fig. 3:**
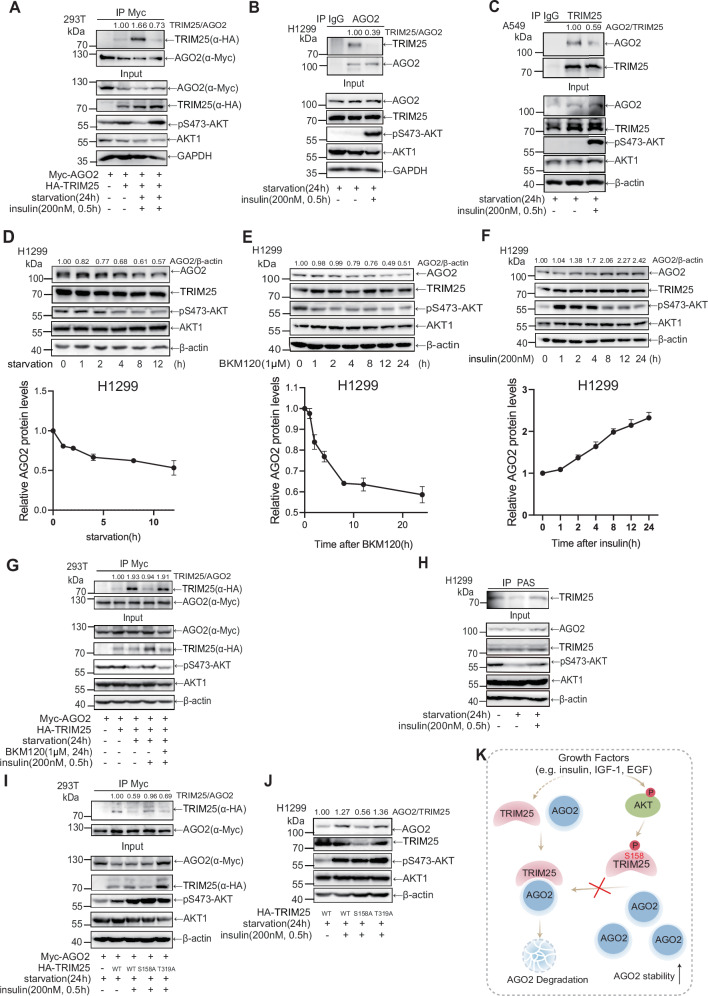
PI3K-AKT signaling stabilizes AGO2 by phosphorylating TRIM25 at S158 to block ubiquitination. **A** 293T cells transfected with Myc-AGO2 and HA-TRIM25 were serum-starved for 24 h and followed by stimulation with insulin for 0.5 h before being harvested. Cell lysates were used for immunoprecipitation with anti-Myc antibody, and then analyzed by western blotting with the indicated antibodies. **B**, **C** H1299 (**B**) and A549 (**C**) cells were serum-starved for 24 h and followed by stimulation with insulin for 0.5 h before being harvested. Cell lysates were used for immunoprecipitation with either anti-AGO2 or anti-TRIM25 antibody, followed by immunoblotting with the indicated antibodies. **D**–**F** The stability of endogenous AGO2 protein was determined in H1299 cells treated with starvation (**D**), BKM120 (**E**), or insulin (**F**) for the indicated period of time. Band intensities were quantified by ImageJ software. **G** 293T cells transfected with Myc-AGO2 and HA-TRIM25 were serum-starved for 24 h and followed by stimulation with insulin for 0.5 h before being harvested. For the BKM120-treated group, BKM120 (1 μM) is added 24 h prior to insulin stimulation. Cell lysates were used for immunoprecipitation with anti-Myc antibody, and then analyzed by western blotting with the indicated antibodies. **H** H1299 cells were serum-starved for 24 h and followed by stimulation with insulin for 0.5 h before being harvested. Phosphorylated proteins recognized by the PAS antibody were pulled down from lysates, and TRIM25 was detected using the specific antibody. **I** 293T cells expressing Myc-AGO2 together with WT or mutant TRIM25 were serum-starved for 24 h, followed by stimulation with insulin for 0.5 h before being harvested. Lysates were subjected to immunoprecipitation with anti-Myc antibody to detect AGO2 interaction with TRIM25. **J** Western blotting analysis for endogenous AGO2 in H1299 cells transfected with HA-TRIM25^WT^, HA-TRIM25^S158A^, or HA-TRIM25^T319A^. Cells were serum-starved for 24 h and followed by stimulation with insulin for 0.5 h before being harvested. Band intensities were quantified by ImageJ software. **K** A working model of PI3K-AKT signaling regulating the TRIM25–AGO2 axis.

To elucidate the upstream signaling pathways regulating TRIM25-mediated AGO2 degradation, we first examined the impact of growth factors on the TRIM25–AGO2 interaction. Co-IP assays in HA-TRIM25- and Myc-AGO2-transfected 293T cells revealed that insulin stimulation (0.5 h) following serum deprivation (24 h) disrupted TRIM25–AGO2 binding (Fig. 3A). This effect was corroborated in endogenous Co-IP experiments using H1299, A549, and DU145 cells (Figs. 3B, C and S3A). Similarly, EGF and IGF-1 treatment dissociated TRIM25 from AGO2 (Fig. S3B, C), suggesting a broad response to growth factor signaling. Next, to determine the functional impact of this regulation on AGO2 stability, we analyzed AGO2 protein levels under different conditions. In serum-starved H1299 cells, AGO2 levels progressively decreased with prolonged starvation, a trend that correlated with declining pS473-AKT levels (Fig. 3D). Similarly, pharmacological inhibition of AKT phosphorylation using the PI3K inhibitor BKM120 (buparlisib) led to time-dependent AGO2 destabilization (Fig. 3E). In contrast, insulin treatment following serum starvation restored AGO2 levels over time (Fig. 3F). These results suggest that growth factor signaling through the PI3K/AKT activation prevents AGO2 degradation by inhibiting TRIM25–AGO2 complex formation.

To determine whether growth factors modulate the TRIM25–AGO2 interaction *via* PI3K-AKT signaling, we employed pharmacological inhibition of PI3K. HEK293T cells co-transfected with Myc-AGO2 and HA-TRIM25 were serum-starved for 24 h, with one group receiving concurrent treatment with the PI3K inhibitor BKM120 (1 μM). Following insulin stimulation (0.5 h), Co-IP analysis revealed that while insulin treatment weakened the TRIM25–AGO2 interaction, this effect was completely blocked by PI3K inhibition (Fig. 3G). These results demonstrate that growth factors regulate the TRIM25–AGO2 complex specifically through the PI3K-AKT pathway.

Phosphorylation frequently modulates protein-protein interactions, thereby regulating ubiquitination and stability [27]. We therefore hypothesized that AKT-mediated phosphorylation of TRIM25 disrupts its binding to AGO2. In serum-starved H1299 cells stimulated with insulin (0.5 h), IP with a phospho-AKT substrate (PAS) antibody showed that TRIM25 phosphorylation decreased during starvation but increased upon insulin treatment (Fig. 3H), suggesting AKT directly phosphorylates TRIM25 to stabilize AGO2.

Sequence analysis identified two conserved AKT phosphorylation motifs in TRIM25—Ser158 (S158) and Thr319 (T319)—both fitting the RXXS*/T* consensus. Phospho-deficient mutants S158A and T319A partially reduced AKT-mediated TRIM25 phosphorylation (Fig. S3D). Functional studies in 293T cells revealed that the S158A mutant enhanced TRIM25–AGO2 binding (Fig. 3I) and accelerated AGO2 degradation (Fig. 3J), whereas T319A had no effect. Structural modeling (AlphaFold) confirmed both sites are solvent-exposed (Fig. S3E), consistent with their regulatory potential. Collectively, these results establish that growth factor-activated PI3K-AKT signaling phosphorylates TRIM25 at S158, dissociating it from AGO2 to block AGO2 degradation (Fig. 3K).

### TRIM25 drives tumor progression *via* AGO2-dependent mechanisms

**Fig. 4:**
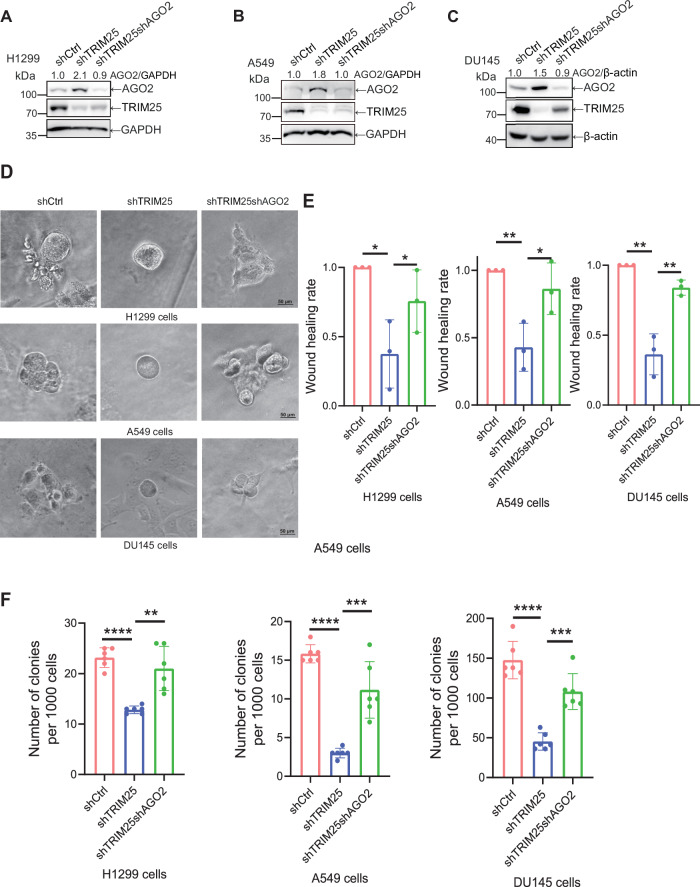
TRIM25 drives tumor progression via AGO2-dependent mechanisms. **A**–**C** Construction of stable cell lines by knocking down AGO2 with shRNA in TRIM25-knockdown H1299 (**A**), A549 (**B**), and DU145 (**C**) cells. Band intensities were quantified by ImageJ software. All the above experiments were repeated at least three times. Quantification of Western blot results and corresponding statistical analyses are presented in Fig. S4A–C. **D** 3D culture assay in H1299, A549, and DU145 cells, photographs were taken 3 days later, scale: 50 μM, *n* = 3 independent experiments. **E** The quantification results of the wound healing assay in stable H1299, A549, and DU145 cells. Data were presented as mean ± SD, *n* = 3 independent experiments. Statistical analysis was performed using a two-tailed unpaired *t*-test. ^*^P < 0.05, ^**^P < 0.01. **F** Statistics of the number in the soft-agar colony formation assay, H1299, A549, and DU145 cells were seeded at a density of 1000/well and cultured for 4 weeks. Data were presented as mean ± SD, *n* = 6 independent experiments. Statistical analysis was performed using a two-tailed unpaired *t*-test. ^**^P < 0.01, ^***^P < 0.001 and ^****^P < 0.0001.

To determine if TRIM25 promotes cancer progression *via* AGO2-dependent mechanisms, we performed AGO2 knockdown in TRIM25-deficient H1299, A549, and DU145 cells (Figs. 4A–C and S4A–C). TRIM25 knockdown impaired vascular mimicry (VM) formation, and this effect was reversed by AGO2 co-depletion (Fig. S4D). In 3D cultures, TRIM25-deficient cells showed reduced invasiveness (smooth colonies with fewer protrusions), while dual TRIM25/AGO2 knockdown restored the scattered, invasive growth pattern of control cells (Fig. 4D). Similarly, TRIM25 knockdown suppressed cell migration (wound-healing assays, Figs. 4E and S4E–G) and anchorage-independent growth (soft-agar assays, Fig. 4F), both of which were rescued by AGO2 depletion. These findings establish AGO2 as the key downstream effector of TRIM25 in promoting tumor progression.

### TRIM25–AGO2 axis may modulate drug resistance through miR-148b-5p-associated ABCC1 regulation

To further investigate the biological implications of the TRIM25–AGO2 axis, we first examined the subcellular localization of TRIM25-mediated AGO2 degradation. Given that AGO2 localizes to multiple cellular compartments, including the cell membrane [28], cytoplasm [29], and nucleus [30], we performed subcellular fractionation experiments using H1299-shTRIM25 stable cell lines. Immunoblot analysis revealed that AGO2 was predominantly cytoplasmic, and TRIM25 knockdown-induced AGO2 accumulation occurred specifically in this compartment, with no significant changes in nuclear or membrane fractions (Fig. S5A, B). This cytoplasmic-specific regulation suggests that TRIM25 primarily controls AGO2 stability in the cytoplasm, where AGO2 executes its main function in RNA interference.

**Fig. 5:**
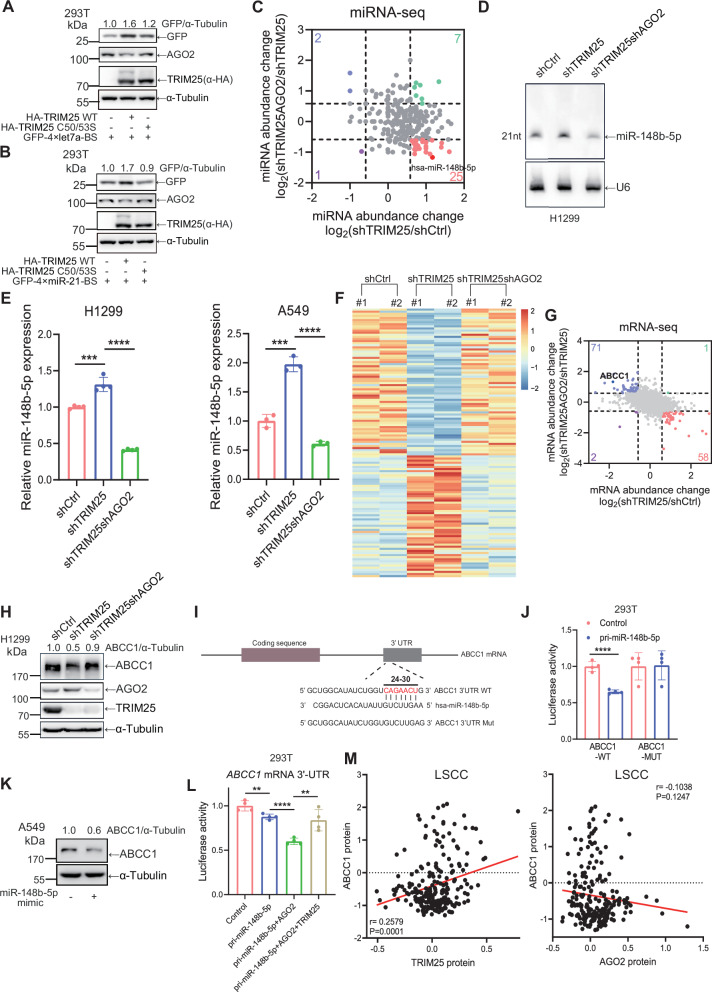
TRIM25–AGO2 axis may modulate drug resistance through miR-148b-5p-associated ABCC1 regulation. **A** 293 T cells co-transfected with GFP-4 × let7a-BS, with HA-TRIM25^WT^ or HA-TRIM25^C50/53S^, were harvested for Western blotting analysis with anti-GFP antibody. **B** 293T cells co-transfected GFP-4 × miR-21-BS, with HA-TRIM25^WT^ or HA-TRIM25^C50/53S^, were harvested for Western blotting analysis with anti-GFP antibody. **C** Scatter plots showing the miRNA expression changes in H1299 stable cell lines. **D** Northern blotting analysis for endogenous miR-148b-5p in H1299 stable cell lines. **E** qPCR for endogenous miR-148b-5p in H1299 and A549 stable cell lines. Data were presented as mean ± SD, *n* = 3 or 4 independent samples. Statistical analysis was performed using a two-tailed unpaired *t*-test, ^***^P < 0.001, ^****^P < 0.0001. **F** Heat map showing the mRNA expression changes in H1299 stable cell lines. 71 mRNAs were downregulated in the shTRIM25 group but upregulated in the shTRIM25/shAGO2 group, whereas 58 mRNAs were upregulated in the shTRIM25 group but downregulated in the shTRIM25/shAGO2 group. **G** Scatter plots showing the mRNA expression changes in H1299 stable cell lines. **H** Western blotting analysis for endogenous ABCC1 in H1299 stable cell lines. **I** The sequences of wild-type/mutant ABCC1 and miR-148b-5p were predicted according to the putative binding sites obtained from TargetScan. **J** Luciferase reporter assay detected the changes in the luciferase activity of wild-type and mutant ABCC1 caused by pri-miR-148b-5p. Data were presented as mean ± SD, *n* = 4. Statistical analysis was performed using a two-tailed unpaired *t*-test, ^****^P < 0.0001. **K** Western blotting analysis for endogenous ABCC1 in A549 cells overexpressing miR-148b-5p mimic. **L** Luciferase reporter assay detected the changes in the luciferase activity of wild-type ABCC1 in 293 T cells overexpressing pri-miR-148b-5p, Myc-AGO2, and HA-TRIM25. Data were presented as mean ± SD, *n* = 4. Statistical analysis was performed using a two-tailed unpaired *t*-test. ^**^P < 0.01, ^***^P < 0.001, ^****^P < 0.0001. **M** Linear regression analysis of the patients with LSCC showed a positive correlation between TRIM25 and ABCC1 and a negative correlation between AGO2 and ABCC1 proteins, pearson coefficient and *P* values were indicated. Band intensities were quantified by ImageJ software.

To assess the functional consequences of TRIM25-mediated AGO2 regulation on miRNA activity, we employed two sensitive GFP reporter systems: GFP-4×miR21-BS (miR-21 reporter) and GFP-4×let-7a-BS (let-7a reporter), each containing four tandem miRNA binding sites in the 3’UTR of GFP [31]. Co-transfection of these reporters with either TRIM25^WT^ or its catalytic mutant TRIM25^C50/53S^ in 293T cells revealed that TRIM25^WT^ overexpression reduced AGO2 protein levels and suppressed both let-7a and miR-21 activity, as indicated by increased GFP expression (Fig. 5A, B). In contrast, TRIM25^C50/53S^ had no effect on AGO2 stability or miRNA-mediated repression, demonstrating that TRIM25’s E3 ligase activity is essential for regulating the AGO2/miRNA pathway.

To comprehensively assess the global regulatory effects of the TRIM25–AGO2 axis on miRNA-mediated gene silencing, we performed parallel miRNA-Seq and mRNA-Seq analyses in H1299 stable cell lines. The miRNA expression profiling revealed that most differentially expressed miRNAs exhibited coordinated changes with AGO2 protein levels (Figs. 5C and S5C), suggesting that TRIM25 knockdown stabilizes miRNAs by increasing AGO2 availability. This observation aligns with established mechanisms of miRNA turnover, wherein miRNAs are rapidly degraded upon dissociation from AGO2 [32, 33]. To validate these findings, we focused on miR-148b-5p, the most significantly altered miRNA in our sequencing data. Both Northern blotting and qPCR analyses confirmed that TRIM25 depletion elevated miR-148b-5p levels, an effect reversed by AGO2 knockdown (Fig. 5D, E), demonstrating AGO2’s essential role in maintaining miR-148b-5p expression. Furthermore, GO pathway enrichment analysis of predicted miR-148b-5p target genes revealed significant enrichment in biological processes related to negative regulation of cell adhesion and migration (Fig. S5D).

In addition to miR-148b-5p, our miRNA-seq data revealed more than twenty other tumor-suppressive miRNAs whose expression is regulated by the TRIM25–AGO2 pathway, including members of the miR-30 family (e.g., miR-30a-5p [34], miR-30e-5p [35]), miR-152-3p [36], miR-26b-5p [37], and miR-186-5p [38] (Fig. S5C). These miRNAs have been reported to inhibit cell proliferation, migration, invasion, or EMT in various cancer types. Consistent with the sequencing results, we experimentally confirmed that miR-186-5p expression changed in the same direction as observed in our miRNA-seq data (Fig. S5E), suggesting that it is also regulated by the TRIM25–AGO2 pathway. These findings suggest that TRIM25 may regulate tumor-related gene expression *via* AGO2, potentially promoting tumor progression.

The mRNA-seq analysis revealed that the shTRIM25/shAGO2 group largely reversed the transcriptional changes induced by shTRIM25 alone (Fig. 5F, G and Supplementary Table S3), underscoring the central role of the TRIM25–AGO2 axis in modulating gene expression networks. GO enrichment analysis of differentially expressed genes (DEGs) linked these changes to key growth regulatory pathways, including cellular responses to amino acid starvation, growth factor stimulation, and xenobiotic stimuli (Fig. S5F). To further dissect the regulatory impact of this axis, we performed KEGG pathway analysis on up- and down-regulated DEGs separately. Strikingly, we found that the TRIM25–AGO2 axis positively regulates ABC transporters (Fig. S5G), suggesting a potential role in drug resistance mechanisms. To functionally validate our sequencing data, we measured ABCC1 expression in stable cell lines and found that TRIM25 knockdown reduced its levels, an effect reversed by AGO2 knockdown (Figs. 5H and S5H), corroborating our transcriptomic findings.

To identify upstream regulators of ABCC1, we integrated miRNA-seq data with TargetScan predictions [39], pinpointing miR-148b-5p as a top candidate (Fig. 5I). Luciferase reporter assays of the *ABCC1* 3’UTR with wild-type (ABCC1-WT) or mutated (ABCC1-Mut) miR-148b-5p binding sites confirmed that miR-148b-5p selectively suppressed the WT reporter, but not the mutant (Fig. 5J), establishing its direct regulation of ABCC1. Consistent with this, miR-148b-5p mimic transfection in A549 cells and pri-miR-148b-5p overexpression in 293T cells significantly reduced ABCC1 protein levels (Figs. 5K and S5I). We next examined whether the TRIM25–AGO2 axis modulates miR-148b-5p-mediated ABCC1 repression. Co-transfection experiments in 293T cells (using psicheck2-ABCC1 3’UTR, pri-miR-148b-5p, Myc-AGO2, and HA-TRIM25) revealed that AGO2 enhanced, while TRIM25 attenuated, miR-148b-5p’s suppressive effect (Fig. 5L). Clinically, ABCC1 protein levels in LSCC patient samples inversely correlated with AGO2 but positively associated with TRIM25 expression (Fig. 5M). Together, these results demonstrate that the TRIM25–AGO2 axis controls ABCC1 expression *via* miR-148b-5p, revealing a mechanistic link to drug resistance in cancer.

### TRIM25–AGO2–miR-148b-5p axis contributes to ABCC1-associated chemoresistance in cancer

**Fig. 6:**
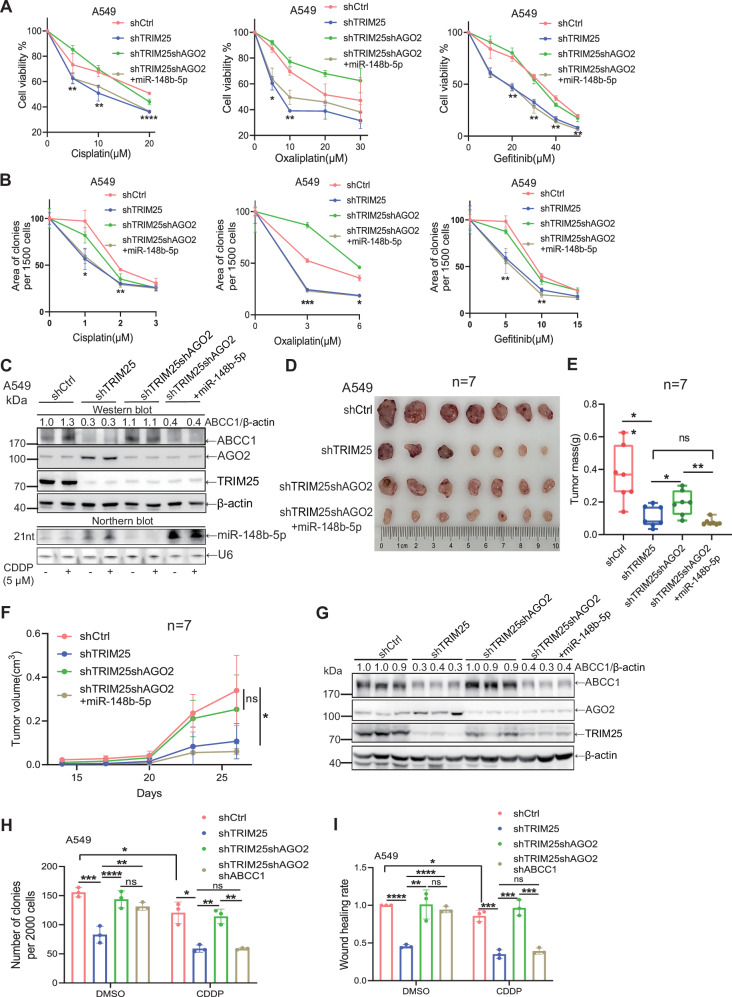
TRIM25–AGO2–miR-148b-5p axis contributes to ABCC1-associated chemoresistance in cancer. **A** CCK8 assay detecting the cell viability of A549 stable cell lines under the indicated Cisplatin, Oxaliplatin, or Gefitinib concentration treatment. Data were presented as mean ± SD, *n* = 3. Statistical analysis was performed using two-way ANOVA. ^*^P < 0.05, ^**^P < 0.01 and ^****^P < 0.0001. **B** Plate colony formation detecting clonogenic ability of A549 stable cell lines under indicated Cisplatin, Oxaliplatin, or Gefitinib concentration treatment. Data were presented as mean ± SD, *n* = 3. Statistical analysis was performed using two-way ANOVA. ^*^P < 0.05, ^**^P < 0.01, and ^***^P < 0.001. **C** A549 cell lines were treated with or without 5 μM Cisplatin for 24 h. Upper, Western blot analysis of ABCC1, AGO2, and TRIM25, with β-actin as a loading control. Lower, Northern blot analysis of miR-148b-5p, with U6 as a loading control. Band intensities were quantified by ImageJ software. **D**–**F** A549 stable cell lines were subcutaneously injected into 6-week-old BALB/c nude mice individually. Mice were sacrificed after 4 weeks of injection. Tumors were dissected (**D**), and tumor weight (**E**) and volume (**F**) were assessed. Data were presented as mean ± SD, n = 7. Statistical analysis was performed using one-way ANOVA. ^*^P < 0.05, ^**^P < 0.01. **G** Western blot for ABCC1, AGO2, and TRIM25 protein levels in 3 randomly selected tumor tissues of nude mice. Band intensities were quantified by ImageJ software. **H**, **I** Effects of ABCC1 knockdown on A549 clonogenic growth and cell migration with or without Cisplatin. The number of colonies in soft agar per 2000 cells (**H**) and wound-healing rates (**I**) were quantified in A549 stable cell lines treated with DMSO or 1 μM Cisplatin. Data are presented as mean ± SD, *n* = 3 independent experiments. Statistical analysis was performed using a two-tailed unpaired Student’s *t*-test; ^*^P < 0.05, ^**^P < 0.01, ^***^P < 0.001, and ^****^P < 0.0001.

Given that the TRIM25–AGO2 axis regulates ABCC1 through miR-148b-5p, we next examined its role in chemoresistance using four stable H1299 and A549 cell lines: shCtrl, shTRIM25, shTRIM25/shAGO2, and shTRIM25/shAGO2 with overexpressing miR-148b-5p expression (Fig. S6A, B). All four lines were treated with increasing concentrations of Cisplatin (CDDP), Oxaliplatin, and Gefitinib, and cell viability and clonogenic survival were assessed by CCK-8 and plate colony-formation assays (Figs. 6A, B and S6C, D). Compared with shCtrl cells, TRIM25 knockdown clearly increased drug sensitivity, whereas additional AGO2 knockdown largely reversed this effect, indicating that AGO2 is required for TRIM25-mediated chemoresistance. Notably, miR-148b-5p overexpression in shTRIM25/shAGO2 cells further reduced cell viability and colony formation across all three drugs, indicating that restoration of miR-148b-5p can resensitize TRIM25/AGO2-deficient cells to chemotherapy.

To directly evaluate the contributions of miR-148b-5p and ABCC1 to TRIM25–AGO2 mediated chemoresistance, we further treated the four stable A549 cell lines with 5 μM CDDP for 24 h and examined ABCC1 and miR-148b-5p expression (Fig. 6C). Western blot analysis revealed high ABCC1 protein levels in shCtrl cells, which decreased upon TRIM25 knockdown, increased after AGO2 knockdown, and were again reduced by miR-148b-5p overexpression. In parallel, Northern blot analysis revealed that miR-148b-5p levels were relatively low in shCtrl cells, elevated in shTRIM25 cells, diminished in shTRIM25/shAGO2 cells, and restored upon miR-148b-5p overexpression, mirroring the pattern observed in untreated cells. These data indicate that CDDP does not substantially change the relative levels of miR-148b-5p and ABCC1 across conditions, which remain primarily governed by the TRIM25–AGO2 axis. Together, these results suggest that miR-148b-5p and ABCC1 may contribute to TRIM25–AGO2-mediated chemoresistance in cancer cells.

To assess the therapeutic potential of miR-148b-5p restoration in vivo, we conducted xenograft experiments by subcutaneously injecting the four A549 cell lines into nude mice. Consistent with the in vitro data, miR-148b-5p-expressing tumors showed markedly slower growth kinetics, with significantly reduced tumor weights and volumes compared to controls (Fig. 6D–F). Western blot analysis of tumor tissues confirmed corresponding changes in ABCC1, AGO2, and TRIM25 protein levels (Fig. 6G). Furthermore, miR-148b-5p overexpression impaired anchorage-independent growth and reduced migration capacity in soft-agar and wound-healing assays (Fig. S6E–G), supporting its role in suppressing tumor progression and chemoresistance downstream of TRIM25–AGO2.

Finally, to determine whether ABCC1 promotes proliferation and migration independently of its drug efflux function, we knocked down ABCC1 in A549 shTRIM25/shAGO2 cells (Fig. S6H) and evaluated anchorage-independent growth using soft-agar colony formation and migration using wound-healing assays. In vehicle (DMSO)-treated conditions, shTRIM25 cells exhibited impaired migration and proliferation compared to shCtrl cells, consistent with Fig. 4E, F. This impairment was rescued by AGO2 knockdown. Notably, ABCC1 knockdown only slightly slowed wound healing and reduced colony formation, suggesting it has no significant effect on baseline cell migration or proliferation (Figs. 6H, I and S6I). However, Cisplatin treatment almost reduced migration and proliferation across all conditions. Moreover, shTRIM25/shAGO2/shABCC1 cells displayed significantly stronger suppression of wound healing and colony survival compared to shTRIM25/shAGO2 cells, with proliferation and migration rates similar to those of shTRIM25 cells (Figs. 6H, I and S6I). These findings align with the established role of ABCC1 in mediating chemoresistance by extruding Cisplatin and other cytotoxic drugs. Collectively, these results demonstrate that the TRIM25–AGO2 axis regulates chemoresistance primarily through miR-148b-5p-dependent modulation of ABCC1, whereas its effects on proliferation and migration likely involve additional miR-148b-5p targets.

### The combination of miR-148b-5p and CDDP enhanced the anti-tumor effect in the NSCLC PDX model

**Fig. 7:**
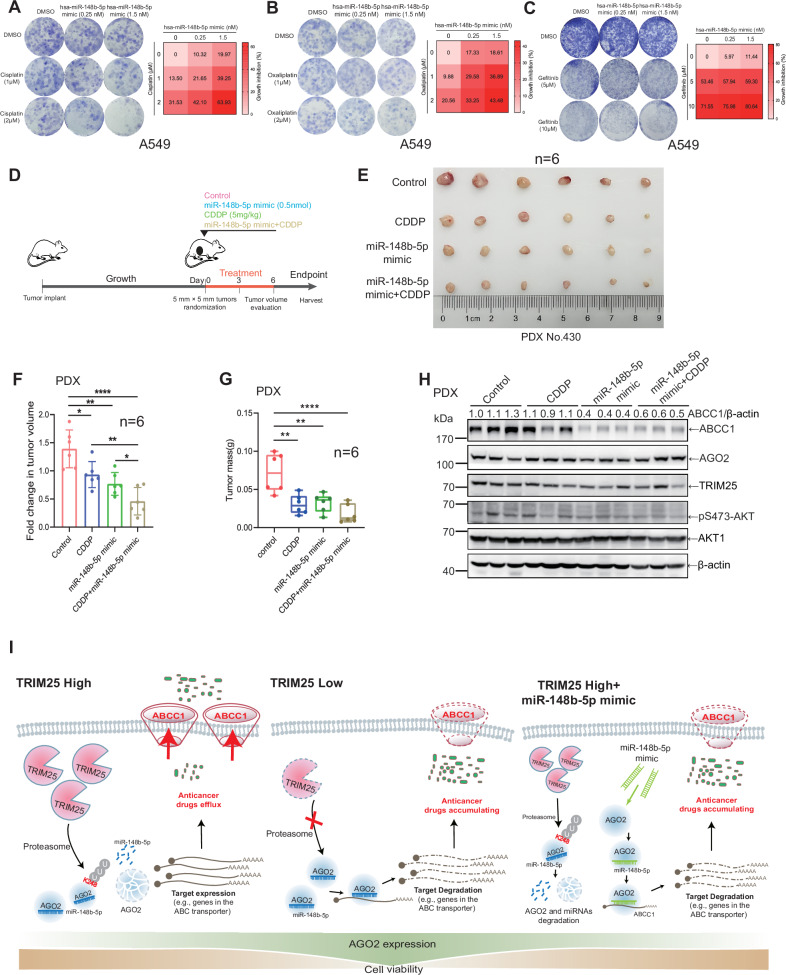
The combination of miR-148b-5p and CDDP enhanced the anti-tumor effect in the NSCLC PDX model. **A**–**C** A549 cells were treated with Cisplatin (**A**), Oxaliplatin (**B**), or Gefitinib (**C**) and miR-148b-5p or their combination at the indicated concentrations. Cells were fixed and stained after 10–12 days. Representative data from three independent experiments. **D** Experimental design of NSCLC PDX model. **E** NSCLC tumor-bearing mice were treated with miR-148b-5p mimic, Cisplatin, and their combination as described in Methods. The tumors were photographed at the end of the experiment. **F** The fold change in tumor volume pre or post treatment was analyzed for NSCLC PDX mice. Data were presented as mean ± SD, *n* = 6. Statistical analysis was performed using one-way ANOVA. ^*^P < 0.05, ^**^P < 0.01, and ^****^P < 0.0001. **G** The tumor weight was measured for NSCLC PDX mice. Data were presented as mean ± SD, *n* = 6. Statistical analysis was performed using one-way ANOVA. ^*^P < 0.05, ^**^P < 0.01, and ^****^P < 0.0001. **H** Western blotting was used to detect the expression levels of ABCC1, AGO2, TRIM25, pS473-AKT, and AKT1 in 3 randomly selected tumor tissues of the NSCLC PDX model. Band intensities were quantified by ImageJ software. **I** A model summarizing that TRIM25 degrades AGO2 to promote chemoresistance by increasing ABCC1 expression. In TRIM25‑high cells, the miR‑148b‑5p mimic restores functional miR‑148b‑5p levels, promotes ABCC1 degradation, and reverses chemoresistance.

To determine if miR-148b-5p is sufficient to enhance chemosensitivity, we expressed miR-148b-5p mimics in A549 shCtrl cells. CCK-8 viability assays showed that miR-148b-5p overexpression significantly sensitized cells to Cisplatin, Oxaliplatin, and Gefitinib. The resulting sensitivity phenocopied that of shTRIM25 cells, whereas the resistance of shTRIM25/shAGO2 cells resembled untransfected controls (Fig. S7A), demonstrating that miR-148b-5p promotes drug-induced cell death. Taken together, our data show that miR-148b-5p overexpression inhibits tumor cell proliferation, migration, and chemoresistance. To evaluate its therapeutic potential, we combined synthetic miR-148b-5p mimic with conventional chemotherapy (Cisplatin, Oxaliplatin, or Gefitinib). Strikingly, colony formation assays revealed a synergistic anti-tumor effect from this combination in both A549 and H1299 cells (Figs. 7A–C and S7B–D), suggesting that miR-148b-5p supplementation could potentiate chemotherapy efficacy in NSCLC.

To further evaluate the clinical relevance of this approach, we employed patient-derived xenograft (PDX) models (Fig. 7D), which better recapitulate the tumor heterogeneity and microenvironment of human NSCLC [40]. For efficient in vivo delivery of miR-148b-5p, we used a protein-based nanocarrier system (CABRi, developed by Guangzhou Glowsi Biotechnology Co., Ltd.; Patent No. 202411527302.4), designed for the targeted delivery of small interfering nucleic acids. Binding efficiency was confirmed by electrophoretic mobility shift assay (EMSA) (Fig. S7E). The CABRi carrier enhanced tumor-selective miR-148b-5p accumulation while minimizing off-target effects. In PDX models, both miR-148b-5p and CDDP monotherapies showed comparable anti-tumor activity, significantly reducing tumor volume and weight compared to controls (Fig. 7E–G). However, the combination therapy demonstrated superior efficacy, achieving greater tumor growth inhibition than either treatment alone. Western blot analysis confirmed that miR-148b-5p reduced ABCC1 expression in tumor tissues. Consistent with the fact that miR-148b-5p specifically targets ABCC1 and not upstream components of the pathway, its overexpression had no obvious effect on the levels of AGO2, TRIM25, pS473-AKT, or total AKT across the four treatment groups (Fig. 7H). We also compared PDX tumors with adjacent non-tumorous lung tissues obtained from lung cancer patients. Two representative tumors from each PDX treatment group were analyzed together with adjacent non-tumorous lung samples. In these comparisons, TRIM25 and ABCC1 are consistently upregulated in PDX tumors. TRIM25 shows no obvious differences among the four PDX treatment groups, whereas ABCC1 levels are specifically reduced in the miR-148b-5p mimic-treated groups. By contrast, AGO2 and pS473-AKT levels are lower in PDX tumors than in the adjacent non-tumorous lung tissues and remain relatively unchanged across the four PDX conditions (Fig. S7F). These data are consistent with our proposed model and support the idea that miR-148b-5p mimicry reverses Cisplatin resistance mainly by downregulating ABCC1 in the PDX model.

These findings suggest that combining conventional chemotherapy with miR-148b-5p may represent a promising therapeutic strategy for NSCLC. By targeting the TRIM25–AGO2–miR-148b-5p–ABCC1 regulatory axis, this approach could overcome limitations of current treatment regimens.

## Discussion

**AGO2**, the core effector protein of the RISC complex, undergoes various post-translational modifications (PTMs) that regulate its activity, subcellular localization, stability, and function, ultimately shaping distinct biological outcomes. The first reported PTM, hydroxylation at proline 700, is critical for AGO2 stability and efficient RNA interference [41]. Ubiquitination of AGO2 has been extensively studied. Mouse Lin-41 (mLin-41/TRIM71) targets AGO2 for ubiquitination to modulate its protein levels in stem cells [42]. Additionally, ZSWIM8 mediates AGO2 polyubiquitination, triggering its degradation via the ubiquitin-proteasome system (UPS) and subsequent miRNA decay [43, 44]. Similarly, STUB1 promotes AGO2 degradation through UPS [45]. Phosphorylation of AGO2 also plays a key regulatory role. AKT3-mediated phosphorylation at serine-387 suppresses mRNA cleavage while enhancing translational repression of miRNA-targeted transcripts [46]. Phosphorylation of Y529, located in AGO2’s small RNA-binding pocket, disrupts small RNA loading. Furthermore, transient phosphorylation of the conserved S824-S834 cluster in the PIWI domain fine-tunes target-binding activity and maintains miRNA-mediated repression efficiency [47]. EGFR- or c-SRC-induced phosphorylation at Y393 impairs DICER binding, inhibiting the maturation of tumor-suppressor-like miRNAs under hypoxia [48, 49]. Other specialized PTMs further diversify AGO2 regulation. Under stress, AGO2 undergoes pADPr modification, enriching it in stress granules and attenuating miRNA-mediated repression and cleavage [50]. *S*-nitrosylation destabilizes AGO2–GW182 interactions, suppressing miRNA function [51, 52]. Malonylation of AGO2 impedes mitochondrial translocation, disrupting mitochondrial translation, elevating oxidative stress, and contributing to diabetic cardiac dysfunction in mice [53].

Our lab discovered that acetylation of AGO2 at K355, K493, and K720 specifically enhances miR-19b maturation [54]. Additionally, Met1-linked linear ubiquitination (M1-Ubi) of AGO2 impairs its ability to load miRNA-targeted mRNAs [31]. More recently, we demonstrated that AGO2 regulates tumor cell resistance to chemotherapy [14], though the underlying mechanism remained unclear. Intriguingly, we observed lower AGO2 expression in tumor tissues compared to normal tissues, raising the question: How is AGO2 protein level regulated? In this study, we addressed this by identifying TRIM25, an E3 ligase highly expressed in tumors, as a key regulator. TRIM25 mediates polyubiquitination at AGO2–K248, targeting AGO2 for proteasomal degradation. Furthermore, we found that this process is controlled by the PI3K-AKT signaling pathway. Our previous work showed that TBK1 phosphorylates AGO2 and affects EGFR-TKI response in NSCLC [14]. Since TBK1 is also an upstream activator of AKT [55], combined with our results that AKT can phosphorylate TRIM25 to regulate AGO2 stability, this indicates that upstream kinase signaling can influence the AGO2–miRNA system through multiple regulatory mechanisms. The final effects likely depend on many factors, including which kinase is active, which site is phosphorylated, the cell type involved, the drug treatment used, and which specific miRNA-mRNA networks are affected. A full understanding of how TBK1, AKT, and other signaling pathways work together to regulate AGO2 in different treatment situations will need more research in future studies. Consistent with our findings, the PI3K/AKT pathway appears largely inactive in the PDX model and unresponsive to Cisplatin treatment, indicating that this signaling cascade is unlikely to drive TRIM25 accumulation or to contribute directly to chemoresistance.

Lung cancer, one of the most frequently diagnosed malignancies, remains the leading cause of cancer-related mortality worldwide [56]. While chemotherapy serves as a first-line treatment [57], most NSCLC patients eventually develop drug resistance. Among various resistance mechanisms, ABC transporter-mediated efflux plays a critical role. Specifically, ABCC1 actively extrudes negatively charged chemotherapeutic agents against concentration gradients, reducing intracellular drug accumulation [58]. Its overexpression is strongly associated with multidrug resistance in cancers [59]. Although several ABCC1 inhibitors have been developed, their clinical application has been limited by toxicity to normal tissues [60–62].

miRNAs function as either oncomiRs that suppress tumor suppressor mRNAs or as tumor suppressor miRNAs that silence oncogenic mRNAs [63]. Notably, miR-148b has been reported to inhibit BRCA progression and metastasis by coordinating a large number of targets [64], and its downregulation is observed in multiple cancers, including NSCLC [65]. Our study reveals that miR-148b-5p directly targets ABCC1, suppressing its expression. Importantly, we demonstrate that the TRIM25–AGO2 axis modulates ABCC1 levels through miR-148b-5p, thereby influencing chemoresistance. Overexpression of miR-148b-5p in lung cancer cells restores chemosensitivity, suggesting a promising strategy to overcome resistance. Mechanistically, we discovered that TRIM25, an E3 ubiquitin ligase, binds AGO2 and induces its polyubiquitination at K248, triggering proteasomal degradation. This TRIM25-mediated AGO2 destabilization promotes drug resistance *via* the AGO2–miR-148b-5p–ABCC1 regulatory axis. Targeting this pathway may provide a novel therapeutic approach for lung cancer treatment.

In addition to its regulation of ABCC1, our data also suggest that miR-148b-5p also influences key genes involved in cell-cycle progression and migration (Supplementary Table S3). Notably, SKP2 [66], a well-known E3 ligase that promotes cell-cycle progression and proliferation, ITGA1 [67], an integrin subunit involved in cell adhesion, invasion, and tumorigenicity, and ZNF704 [68], a biomarker associated with poor prognosis. The coordinated suppression of these factors aligns with the observed reduction in proliferation and migration following miR-148b-5p overexpression. These findings support the idea that miR-148b-5p not only regulates ABCC1-mediated drug efflux, but may also impede NSCLC progression by modulating regulators of the cell cycle and cell motility. Furthermore, our miRNA-seq analysis revealed that the TRIM25–AGO2 pathway also affects additional tumor-suppressive miRNAs that have been reported to inhibit proliferation, migration, invasion, or EMT in various cancers. Together, these results point to a broader regulatory network in which TRIM25-dependent AGO2 degradation regulates a set of tumor-suppressive miRNAs and their downstream targets.

Collectively, these findings suggest that tumors exhibiting a high-TRIM25, low-AGO2, low-miR-148b-5p, and high-ABCC1 profile are likely more aggressive and chemoresistant, and may therefore benefit from treatment incorporating a miR-148b-5p mimic. Importantly, miR-148b-5p overexpression successfully reduced ABCC1 and restored Cisplatin sensitivity even in the absence of TRIM25 and AGO2. This indicates that tumors with the simpler signature of low miR-148b-5p and high ABCC1 could also respond to miR-148b-5p-based therapy. These observations position the miR-148b-5p mimic as a promising strategy to counteract ABCC1-mediated chemoresistance in a subset of NSCLC, while highlighting the broader TRIM25–AGO2–miRNA axis as a target for future combination therapies.

## Materials and methods

### Cell culture and transfection

The human HEK-293T, HEK-293FT, H1299, A549, and DU145 cell lines were obtained from the National Collection of Authenticated Cell Cultures, Shanghai, China. All these cell lines were cultured in Dulbecco’s modified Eagle’s medium (DMEM, Corning) supplemented with 1% penicillin/streptomycin (YEASEN) and 10% fetal bovine serum (FBS, YEASEN) at 37 °C in a humidified incubator with 5% CO_2_. All stable knockdown cell lines, as well as overexpressing cell lines, were generated by lentiviral infection. For transient expression, cell transfection was performed with Lipofectamine 2000 (Invitrogen) or polyethylenimine linear (PEI).

### Antibodies and reagents

Antibodies against AGO2 (#2897), AKT1 (#2938), pS473-AKT (#4060), phospho-Akt substrate (#9611), and Myc (#2276) were purchased from Cell Signaling Technology. Antibodies against ABCC1(#JA93-03) were purchased from HUABIO. Antibodies against GAPDH (#60004-1-Ig), β-Actin (#60008-1-Ig), His-Tag (#66005-1-Ig), α-Tubulin (#66031-1-Ig), GFP(#50430-2-AP), GST (#66001-2-Ig), TRIM25 (#12573-1-AP) and ubiquitin (#10201-2-AP) were purchased from ProteinTech Group. Monoclonal anti-HA antibody (#MMS-101R) was from Covance. Monoclonal anti-Flag antibody was from Sigma-Aldrich. Antibodies against normal mouse IgG (#sc-2025) and normal rabbit IgG (#sc-2027) were purchased from Santa Cruz Biotechnology. Protein A/G agarose beads (#IP05) were purchased from Calbiochem. Polybrene (#H9268) and cycloheximide (#C7698) were purchased from Sigma-Aldrich. Gefitinib (#S1025) and CDDP(#S1166) were purchased from Selleck. Puromycin (#58-58-2) and cell counting kit (CCK8) (40203ES76) were from YEASEN. MG132(#T510313) was from Sangon. All reagents used in this study are listed in the supplement (Supplementary Table S1).

### Plasmids

Human TRIM25 was constructed by PCR-based amplification of cDNA from HEK293T cells, and then subcloned into the pCMV-HA, pCMV-Myc, and pET-28a, respectively. Plasmids pCS2-Myc6-AGO2, pCMV-HA-AGO2, and GST-AGO2 are previously described [31, 54]. Point mutations for AGO2 and TRIM25 were generated by using the KOD-plus-mutagenesis Kit (TOYOBO). The short hairpin RNA (shRNA) oligos for AGO2 and TRIM25 were subcloned into the lentiviral vector pLKO.1, building stable cell lines with the packaging plasmids pMD2G and pCMV-dR8. The sequences of all plasmids were verified by sequencing. The primary miRNA pri-miR-148b-5p was cloned into the pCD513B vector. The ABCC1 3’UTR was cloned into the psiCHECK2 vector. Mutations of ABCC1 3’UTR were sub-cloned by using the KOD-plus-mutagenesis Kit (TOYOBO). Sequences for primers used for plasmid construction and shRNAs were listed in the supplement (Supplementary Table S2).

### Co-IP

HEK293T cells transfected with the indicated plasmids were lysed in ice-cold RIPA buffer (50 mM Tris-HCl, pH 7.4, 150 mM NaCl, 1% NP-40, 1 mM EDTA, and 0.05% SDS) with protease inhibitor cocktail, sonicated, and cleared by centrifugation. The supernatants were immunoprecipitated with Protein A/G agarose beads and antibodies at 4 °C overnight, and then washed at least three times with RIPA buffer without protease inhibitor cocktail, followed by Western blotting analysis.

### Western blotting analysis

Samples were separated by SDS-PAGE and then transferred to a PVDF membrane. After blocking in TBS containing 0.1% Tween 20 and 5% skimmed milk, the membrane was incubated with primary antibodies, followed by incubation with horseradish peroxidase-conjugated secondary antibody. Immunoreactive proteins were visualized using enhanced chemiluminescence.

### Northern blotting analysis

The northern blotting analysis of RNA was conducted as described before [14, 54, 69]. Total RNAs extracted by TRIzol reagent (Invitrogen) from cells were denatured at 95 °C for 5 min. Then, RNAs were fractionated by electrophoresis on the 20% polyacrylamide 8 M urea gel and transferred to the nylon membrane (Roche). After cross-linking, the membrane was pre-hybridized by North2South® Hybridization Buffer at 55 °C for 30 min, and then was hybridized with biotinylated probe within North2South® Hybridization Buffer at 55 °C overnight; and following washed with North2South® Hybridization Stringency Wash Buffer (1×) 15 min at 55 °C for twice times, then incubated within streptavidin-HRP soluted blocking Buffer for 1 h at room temperature, and washed the membrane by wash buffer 5 min for four times and by substrate equilibration buffer 5 min for once, respectively. Finally, the signaling on the membrane was detected by using the Amersham Imager 600 (GE) instrument. Probes used for Northern blotting are listed in the supplement (Supplementary Table S2).

### Protein purification

This method has been previously described [41, 70]. The prokaryotic expression constructs pEGX-4T1-AGO2 and pET-28a-TRIM25 were transformed into BL21 competent cells with 0.5 mM isopropyl β-d-1-thiogalactopyranoside (IPTG) induction for 12–16 h at 16 °C. For GST-AGO2 purification, the bacterial pellet was resuspended in buffer (50 mM Tris-HCl, pH 7.4, 150 mM NaCl) and sonicated. After centrifuging at maximum speed (17,000×*g*) for 20 min at 4 °C, the bacterial solution was passed through a filter column containing GST-Sefinose Resin (Sangon) to capture the GST-protein, and then eluted with GSH buffer (50 mM Tris-HCl, pH 8.0, 20 mM GSH). For His-TRIM25 purification, bacteria were harvested and lysed by sonication in the buffer consisting of 50 mM Tris-HCl, pH 8.0, 150 mM NaCl. The inclusion bodies were harvested by centrifugation at 17,000×*g* for 20 min at 4 °C. After washing twice with 20 mL ice-cold buffer consisting of 2 M urea, 50 mM Tris-HCl (pH 8.0), and 300 mM NaCl, the inclusion bodies were dissolved in 10 mL buffer consisting of 6 M urea, 50 mM Tris-HCl (pH 8.0), and 300 mM NaCl. The denatured proteins were refolded after being dialyzed twice in 2 L ice-cold buffer containing 50 mM Tris-HCl (pH 8.0) and 150 mM NaCl. The purified protein was detected by Coomassie brilliant blue staining and western blotting.

### qRT-PCR analysis

The method for miRNA qRT-PCR was previously described [31]. Briefly, RNAs from cell lysates were extracted using Trizol (Sigma-Aldrich), treated with DNase I (Thermo) to degrade genomic DNA. For miRNA detection, specific miRNA reverse primers and U6 reverse primer were used to reverse transcribe mature miRNAs and U6 snRNA, respectively. Quantitative real-time PCR was performed with qPCR SYBR Green Master Mix (Vazyme) to analyze the indicated RNA abundance. U6 snRNA was used for normalization of mature miRNA. Primers used for qRT-PCR were listed in the supplement (Supplementary Table S2).

### GFP expression for miRSIC activity assay

pEGFP-C1-4xlet-7a-BS and pEGFP-C1-4xmiR21-BS plasmids were constructed as described before [31]. The 293T cells were transfected with pEGFP-C1-4xlet-7a-BS or pEGFP-C1-4xmiR21-BS and indicated plasmids. 48 h after transfection, cells were collected for Western blotting analyses of GFP in protein level.

### Luciferase reporter assay

The *ABCC1* fragment covering miR-148b-5p binding site (wild type and mutant) was used to construct ABCC1-WT/Mut vectors using the psiCHECK2 vector. The acquired vectors were cotransfected with pri-miR-148b-5p or indicated plasmids into 293T cells. After 48 h of cotransfection, the cells were subjected to the dual-luciferase reporter assay according to the manufacturer’s instructions.

### Cellular fractionation

Extraction of cytoplasmic and nuclear proteins was performed using the Nuclear/Cytosol Fractionation Kit (#266-100, BioVision) according to its instructions. Extraction of cytoplasmic and membrane proteins was performed using the membrane/Cytosol Fractionation Kit (#20127ES60, YEASEN) according to its instructions.

### Cell viability

For the cell viability assay, cells were seeded at a density of 1 × 10^4^ on 96-well plates, and treated with CDDP, Gefitinib or Oxaliplatin at the indicated concentration for 48 h. After treatment, the cellular activity was measured by the Cell Counting Kit-8 kit (YEASEN) following the manufacturer’s instructions. The relative viability of cells was analyzed by the ratio of absorbance under drug treatment to no treatment.

### Plate colony formation assay

Stable cell lines were seeded at a density of 1500 cells on 12-well plates. Cells were treated with CDDP, Gefitinib, or Oxaliplatin at the indicated concentration. After 7–10 days, colonies were stained with 0.1% crystal violet overnight, and the number of colonies was counted using ImageJ.

### Soft-Agar colony formation assay

The soft-agar colony formation assay was performed as previously described [71]. Briefly, stable cells were suspended in 2 mL colony formation gel (2 × medium, 10% FBS, 1% penicillin/streptomycin, and 3.5% agar gel), and added to 6-well plates coated with 2 mL base gel (2 × medium, 10% FBS, 1% penicillin/streptomycin, and 0.6% agar gel). After 3–4 weeks, gels were stained with 0.005% crystal violet overnight, and the number of colonies was counted using ImageJ.

### Wound healing assay

For the wound-healing assay, the stable cell lines were seeded in Culture-Insert 2 Well in μ-Dish (iBidi) and cultured overnight. Then, the insert was removed, and cells were washed with PBS. Cells were incubated with serum-free medium and cultured in 5% CO_2_ humidified incubator at 37 °C. Photographs were taken at the indicated times until the wound was healed.

### Vasculogenic mimicry (VM) formation

For the VM assay, matrigel matrix pre-thawed at 4 °C was added into the inner well of μ-slides and incubated for at least 30 min at 37 °C until polymerization. Five thousand cells were added onto the polymerized matrix. Microscopy images were taken after 12 h.

### EMSA

Four pmol of Biotin-labeled RNAs were used for each EMSA reaction. Protein-RNA incubation was carried out with the indicated amount of purified protein CABRi and Biotin-labeled RNA in PBS at room temperature for 30 min. The binding reactions were then mixed with 5 × EMSA loading buffer (75% Glycerol, 2.5 × TBE, 0.06% bromophenol blue, 0.06% xylene cyanol) and loaded onto a 7% native page gel for electrophoresis. After that, the separated protein-RNA was transferred onto the Nylon membrane (Sigma-Aldrich), fixed to the membrane by UV crosslinking (480 mJ/cm^2^). The membrane was then incubated with Stabilized streptavidin-HRP Conjugate (Thermo Fish Scientific) at room temperature for 1 h, washed 3 times for 10 min with 1 × Washing buffer (Thermo Fish Scientific). The signal of Biotin-labeled RNA was detected by Amersham Imager (GE HealthCare).

### Xenograft tumor model

Five-week-old male nude mice were purchased from Shanghai Lingchang Biotechnology Co., Ltd. and maintained in a pathogen-free, temperature-controlled environment with a 12 h light/dark cycle. The experiment of the xenograft tumor model was established as described previously [72, 73]. Stable A549 cell lines were injected subcutaneously into nude mice (*n* = 7) at the final concentration of 4 × 10^6^ cells. A Vernier caliper was utilized to monitor the length and width of the tumors every 3 days, and tumor volume was computed with the following formula: Volume (mm^3^) = length × width^2^/2. Mice were sacrificed 4 weeks later, and tumors were weighed and photographed. All animal experiments were conducted in accordance with the Guidelines for the Care and Use of Laboratory Animals and approved by the Institutional Animal Care and Use Committee of Shanghai Jiao Tong University School of Medicine.

### Patient-derived tumor xenograft (PDX) model

To establish the Non-small Cell Lung Cancer (NSCLC) PDX models, NSCLC tissues were purchased from Nanchang Royo Biotech Co., Ltd. The tumor was cut into 2 mm × 2 mm × 2 mm (8 mm^3^) sections, which were subcutaneously inoculated into the scapular region of 6-weeks-old male nude mice. Once tumors reached an average of 5 mm × 5 mm in size, mice were assigned at random to four clusters. Mice were treated with miR-148b-5p mimic (0.5 nmol) by tail vein injection, with/without CDDP (5 mg/kg) by intraperitoneal injection every 3 days, with 3 injections in total. On day 3 after the final treatment, mice were euthanized by cervical dislocation, and xenograft tumors were subsequently separated and photographed with a camera. The patient had signed the informed consent form, and the sample collection was approved by the medical ethics committee of the Second Affiliated Hospital of Nanchang University (Permit No. 2021012).

### High-throughput sequencing for miRNA-Seq and RNA-Seq

For miRNA-Seq, total RNA was extracted from stable cell lines using a TRIZOL reagent. Extracted RNA was used for preparing the miRNA sequencing library, which included 30-adapter ligation, 50-adapter ligation, cDNA synthesis, and PCR amplification and size selection of ~ 150 bp PCR amplicons (corresponding to ~22 nt miRNAs). The libraries were denatured as single-stranded DNA molecules, captured on Illumina flow cells, amplified in situ as clusters, and finally sequenced for 50 cycles on an Illumina HiSeq sequencer following the manufacturer’s instructions.

For RNA-Seq, total RNA extracted from the indicated stable cell lines by TRIZOL reagent was used for removing the rRNAs with the NEBNext rRNA Depletion Kit (New England Biolabs) according to the manufacturer’s instructions. The rRNA-depleted RNAs were constructed into RNA sequencing libraries by using NEBNext Ultra II Directional RNA Library Prep Kit (New England Biolabs, Inc., Massachusetts, USA) according to the manufacturer’s instructions. RNA-Seq libraries were controlled for quality and quantified using the BioAnalyzer 2100 system (Agilent Technologies, Inc., USA), and the libraries' sequencing was performed on an Illumina HiSeq instrument with 150 bp paired-end reads. High-throughput sequencing for RNA-Seq and miRNA-Seq was all done by Cloud-Seq Biotech (Shanghai, China).

### Analyses for high-throughput sequencing data

For miRNA-Seq, raw data were generated after sequencing, image analysis, base calling, and quality filtering on an Illumina sequencer, and finally quality controlled by Q30. The adapter sequences were trimmed, and the adapter-trimmed-reads (≥15 nt) were left by the cutadapt software (version 1.9.2). Then the trimmed reads from all samples were pooled, and miRDeep2 software (version 2.0.0.5) was used to predict novel miRNAs. The trimmed reads were aligned to the merged human miRNA databases using Novoalign software (version 3.02.12) with at most one mismatch. The number of mature miRNA mapped tags was defined as the raw expression levels of that miRNA. The read counts were normalized by the TPM (tag counts per million aligned miRNAs) approach. Differentially expressed miRNAs between the two samples were filtered through Fold change.

For RNA-Seq, paired-end reads were harvested from the Illumina HiSeq 4000 sequencer, and were quality controlled by Q30. After 3′ adapter trimming and low-quality reads removal by the cutadapt software (v1.9.3). The high-quality reads were aligned to the human reference genome (UCSC hg38) with hisat2 software (v2.0.4). Then, guided by the Ensembl gtf gene annotation file, cuffdiff software (v2.2.1, part of cufflinks) was used to get the Fragments Per Kilobase of exon model per Million mapped fragments (FPKM) as the expression profiles of mRNA, and fold change was calculated based on FPKM, differentially expressed mRNA were identified.

### MS analysis

To identify potential interacting proteins of AGO2, 293T cells transiently expressing Flag-AGO2 were lysed with RIPA lysis buffer (50 mM Tris-HCl, pH 7.4, 150 mM NaCl, 1% NP40, 0.05% SDS, 1 mM EDTA, 1% protease inhibitor cocktail). The lysates were incubated with anti-Flag antibody and protein A/G-agarose beads at 4 °C overnight. Beads were washed five times with the same RIPA lysis buffer and then dissolved in 10% SDS lysis buffer at 95 °C for 10 min. IP efficiency was validated by Western blotting, and the dissolved samples were directly used in the trypsin digestion step.

### Statistical analysis

The investigator was blinded to group allocation during outcome assessment to minimize bias. Statistical analyses were performed with GRAPHPAD PRISM 8 (Graphpad Software, Boston, MA, USA). Data in this work were presented as the mean ± standard deviation (*n* ≥ 3). All data were tested for normality, and the appropriate statistical tests were selected based on the distribution and experimental design. Homogeneity of variance was assessed; for two-group comparisons, an *F*-test was used. Statistical evaluations between two groups were performed by a two-tailed unpaired *t*-test. Experiments with more than three groups were evaluated by one-way or two-way ANOVA. ^*^P < 0.05, ^**^P < 0.01, ^***^P < 0.001, and ^****^P < 0.0001 were considered statistically significant.

## Supplementary information

Supplementary Figure S1-7

Supplementary Table S1-S2

Supplementary Table S3

Uncropped original western blots

## Data Availability

All sequencing data generated in this study have been deposited at GEO and are publicly accessible under accession numbers GSE290308. The MS proteomics data have been deposited to the ProteomeXchange Consortium (https://proteomecentral.proteomexchange.org) *via* the iProX partner repository [74, 75] with the dataset identifier PXD062830. All materials used in this study are available upon request.
