# Supplementary material for: Targeting the TRIM25–AGO2–miR-148b-5p–ABCC1 axis overcomes chemoresistance in non-small cell lung cancer
Source: Cell Death Dis. 2026 Apr 30;17(1):579. doi: 10.1038/s41419-026-08802-1 (PMC13276176; doi:10.1038/s41419-026-08802-1)
Supplement: Supplementary file 1 — Supplementary Figure S1-7 [file 41419_2026_8802_MOESM1_ESM.pdf]

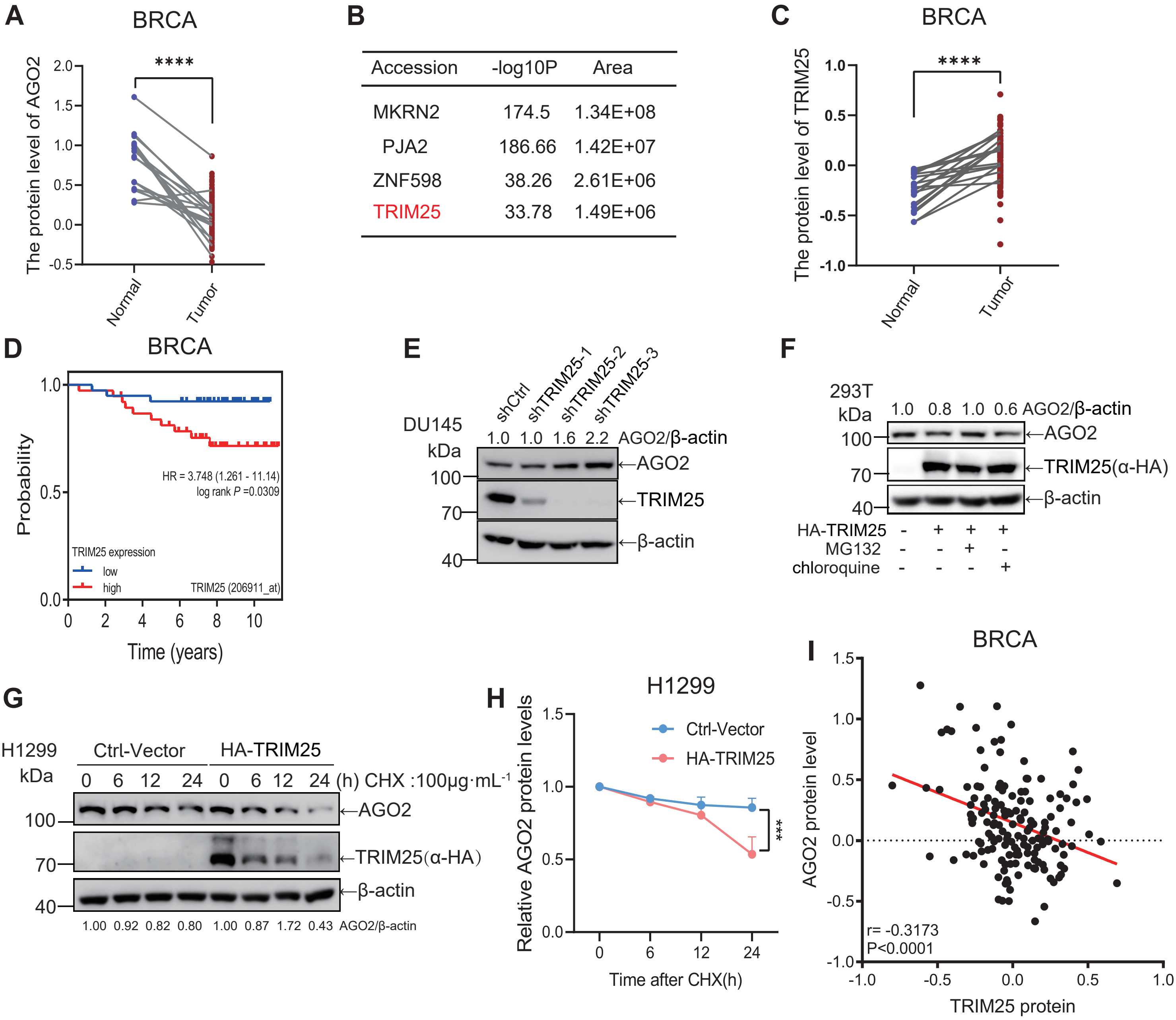

Figure S1. TRIM25 negatively regulates AGO2 protein levels in cancer cells. A Protein levels of AGO2 were deregulated in BRCA. Statistical analysis was performed using two-tailed unpaired t-test, **** $P < 0.0001$. **B** A list of AGO2-binding proteins was identified through mass spectrometric analysis. HEK293T cells expressing Flag-AGO2 were generated, and AGO2 complexes were subjected to mass spectrometric analysis. **C** Protein levels of TRIM25 were deregulated in BRCA.Statistical analysis was performed using two-tailed unpaired t-test, **** $P < 0.0001$. **D** Kaplan-Meier survival curves comparing overall survival between patients with high (red) and low (blue) TRIM25 expression levels in BRCA (PrognoScan microarray data). **E** Western blotting analysis for endogenous AGO2, TRIM25 in DU145 stable cells with TRIM25 knockdown. **F** HA-TRIM25 was transfected into 293T cells, followed by the treatment with MG132 (20 μM) or chloroquine (10 μM) for 4 h. **G, H** The half-life of endogenous AGO2 protein was determined in TRIM25 overexpressed H1299 cells with treatment of CHX (100 $\mu g \cdot mL^{-1}$) for the indicated time points **(G)**, and quantified and normalized to β-actin with Image J **(H)**. MG132 pretreatment prior to CHX to normalize protein levels. Data were presented as means ± SD, n = 3. Statistical analysis was performed using one-way ANOVA. *** $p < 0.001$.**I** Linear regression analysis of the patients with BRCA showed a significant negative correlation between AGO2 and TRIM25 proteins, pearson coefficient and P values were indicated. Band intensities were quantified by ImageJ software.

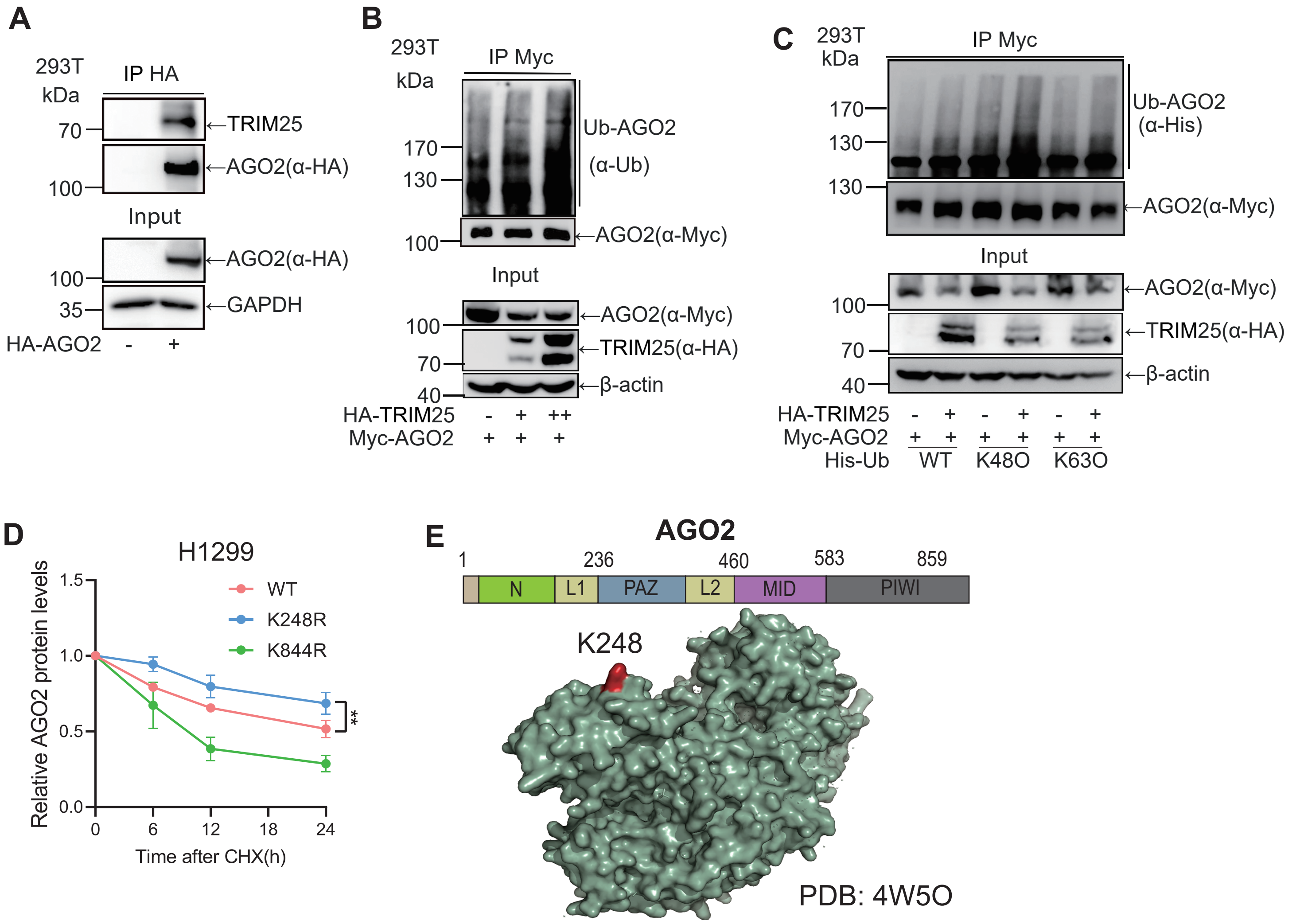

Figure S2. TRIM25 binds and ubiquitinates AGO2 at K248 to drive proteasomal degradation. A HA-AGO2 was transfected into 293T cells, followed by immunoprecipitation with the HA antibody and immunoblotting analysis with TRIM25 antibody. **B** Lysates from 293T cells transfected with Myc-AGO2 and an increasing amount of TRIM25 were immunoprecipitated with anti-Myc antibody, and followed by western blotting analysis with anti-ubiquitin antibody**. C** 293T cells were transfected with Myc-AGO2 and His-Ub (WT, k48 only or k63 only) with or without HA-TRIM25, followed by immunoprecipitation with the anti-Myc antibody, and probed with the anti-His antibody. **D** Quantification result of AGO2 intensity in Fig.2J, n=3. **E** The spatial conformation of the K248 site (PDB: 4W5O).

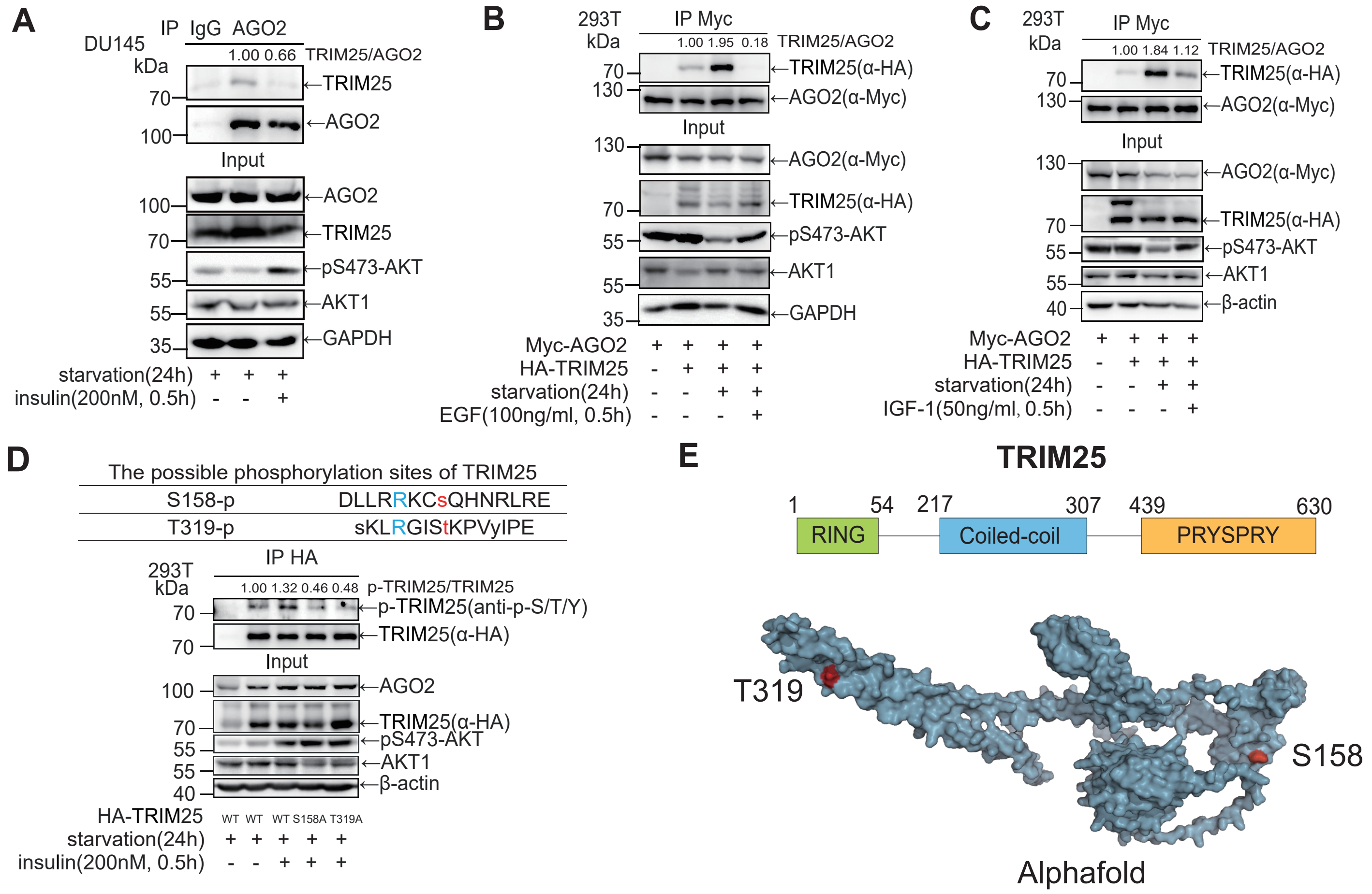

Figure S3. PI3K-AKT signaling stabilizes AGO2 by phosphorylating TRIM25 at S158 to block ubiquitination. **A** DU145 cells were serum-starved for 24 h and followed by stimulation with insulin for 0.5 h before harvested. Cell lysates were used for immunoprecipitation with anti-AGO2 antibody, followed by immunoblotting with as indicated antibodies. **B, C** 293T cells transfected with Myc-AGO2 and HA-TRIM25 were serum-starved for 24 h and followed by stimulation with EGF **(B)** or IGF-1 for 0.5 h **(C)** before harvested. Cell lysates were used for immunoprecipitation with anti-Myc antibody, and then analyzed by western blotting with as indicated antibodies. **D** WT HA-TRIM25, mutant HA-TRIM25^{S158A} and HA-TRIM25^{T319A} were expressed in 293T cells that were serum-starved for 24 h and followed by stimulation with insulin for 0.5 h. Lysates were immunoprecipitated with anti-TRIM25 antibody, and then immunoblotted by anti-phospho-S/T/Y antibody. **E** The S158 and T319 sites were predicted by Alpha Fold2 and showed by PyMol.

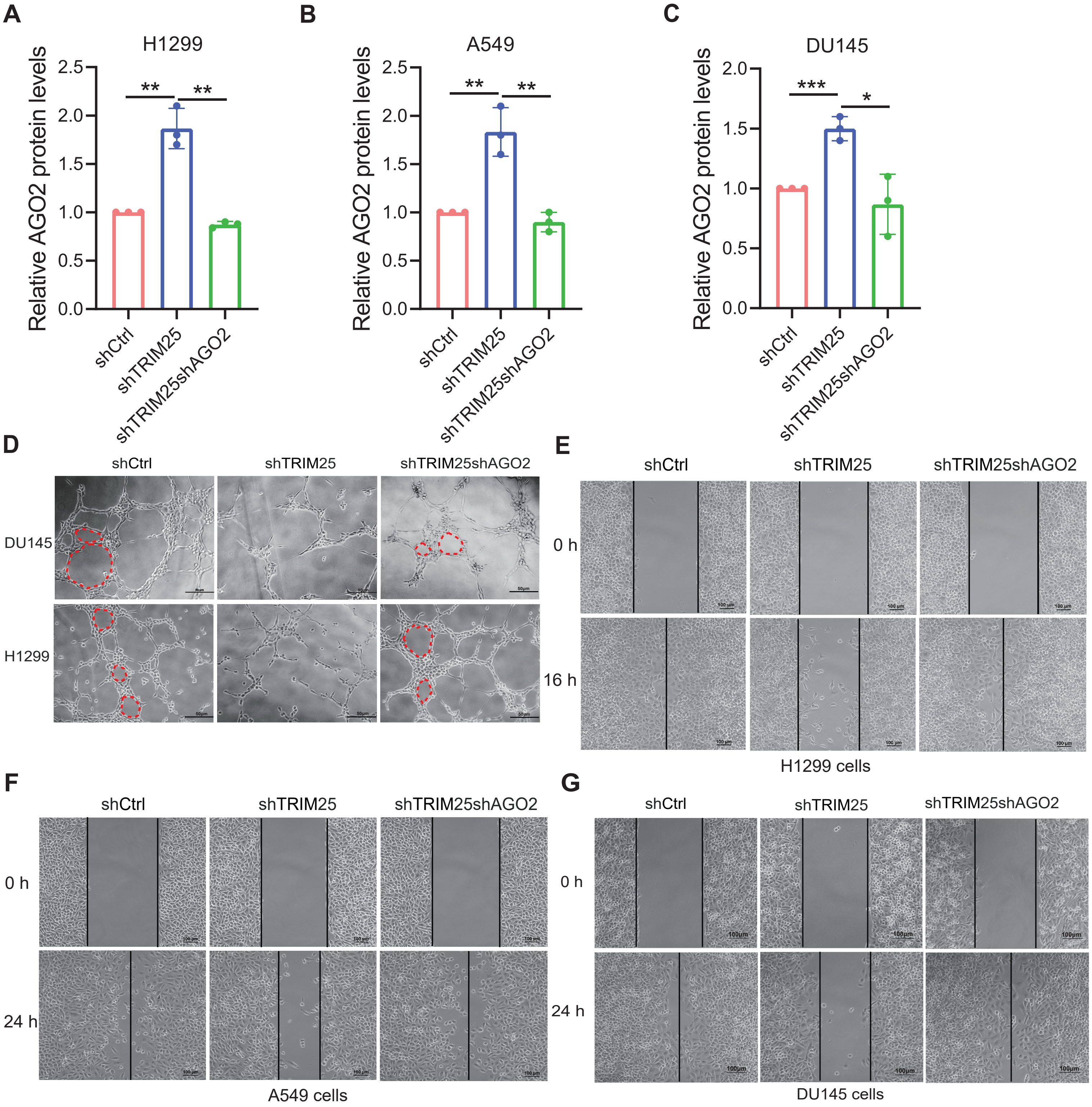

Figure S4. Knockdown of TRIM25 inhibits cancer progression by upregulating AGO2. A-C Quantification results of AGO2 intensity in Fig.4A-C. Data are presented as mean ± SD, n = 3 independent experiments. Statistical analysis was performed using a two-tailed unpaired Student's t-test; $^*P < 0.05$, $^{**}P < 0.01$, $^{***}P < 0.001$. **D** Vasculogenic mimicry assays of DU145 and H1299 stable cell lines. Representative pictures were taken 12 h later. scale: 100 μm. **E-G** Wound healing assay, photographs were taken at 0 h and 16 or 24 h later in H1299, A549 and DU145 cells, scale: 100 μm. All above experiments were repeated at least 3 times.

A

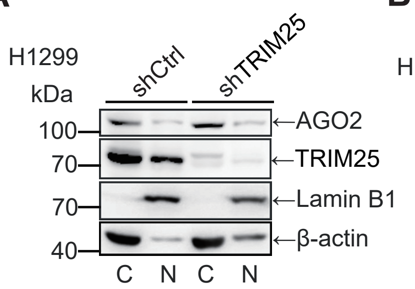

B

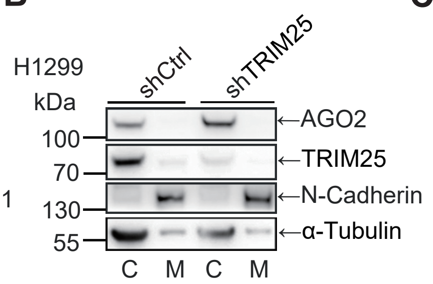

C

mature.miRNA	log2(shTRIM25 /shCtrl)	log2(shTRIM25shAGO2 /shTRIM25)
hsa-miR-30a-5p	1.610524808	-0.985971055
hsa-miR-30e-5p	1.411426246	-0.703606997
hsa-miR-23a-3p	1.401098308	-0.841010476
hsa-miR-148b-5p	1.169925001	-1.169925001
hsa-miR-147b-5p	1.157541277	-0.610053482
hsa-miR-30d-5p	1.105353	-1.062284278
hsa-miR-1305	1	-1
hsa-miR-1306-5p	1	-0.678071905
hsa-miR-152-3p	1	-0.807354922
hsa-miR-196a-5p	1	-1
hsa-miR-3912-3p	1	-0.678071905
hsa-miR-421	1	-0.874469118
hsa-miR-17-5p	0.906890596	-0.600229257
hsa-miR-767-5p	0.906890596	-0.658963082
hsa-miR-26b-5p	0.823969636	-0.883675882
hsa-miR-660-5p	0.788495895	-0.788495895
hsa-miR-181d-5p	0.736965594	-0.906890596
hsa-miR-18a-5p	0.736965594	-0.621488377
hsa-miR-454-3p	0.736965594	-0.736965594
hsa-miR-93-5p	0.725560831	-0.79538228
hsa-miR-425-5p	0.707819248	-0.637429921
hsa-miR-186-5p	0.700439718	-1.056583528
hsa-miR-24-3p	0.696837482	-0.907584366
hsa-miR-148a-3p	0.678744647	-0.708660225
hsa-miR-330-3p	0.632268215	-0.632268215

D

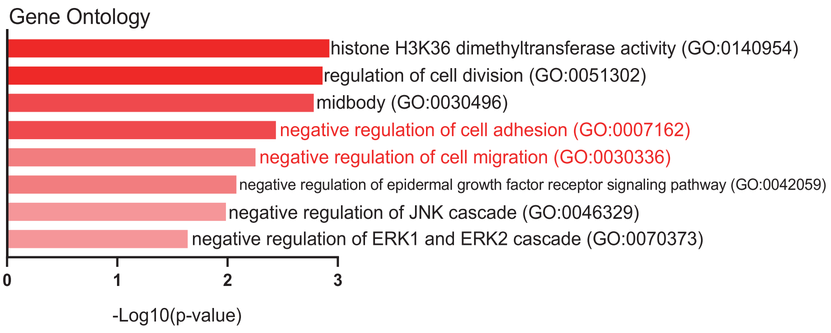

E

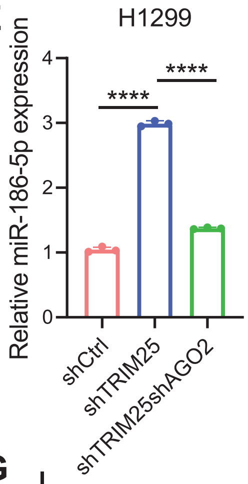

F

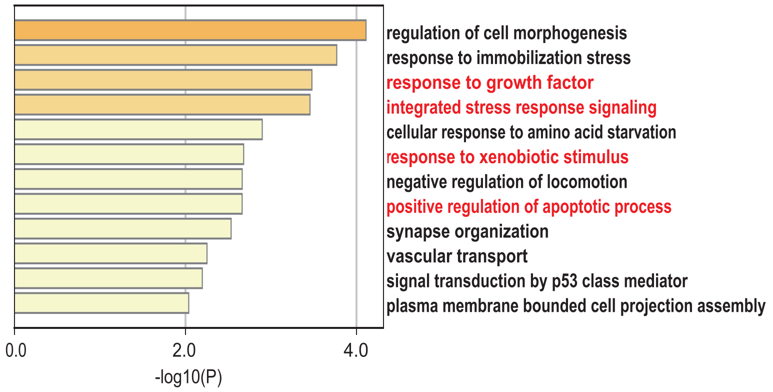

G

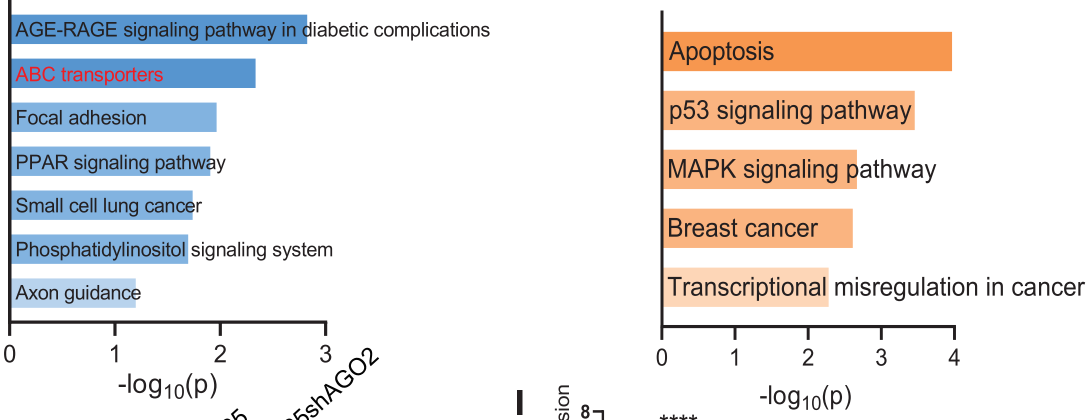

H

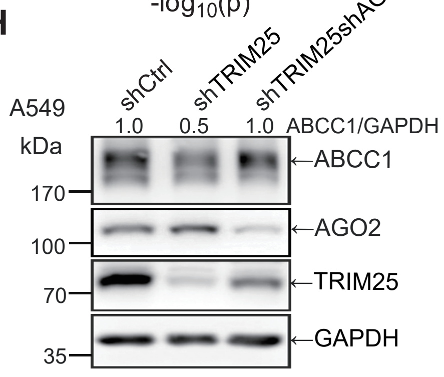

I

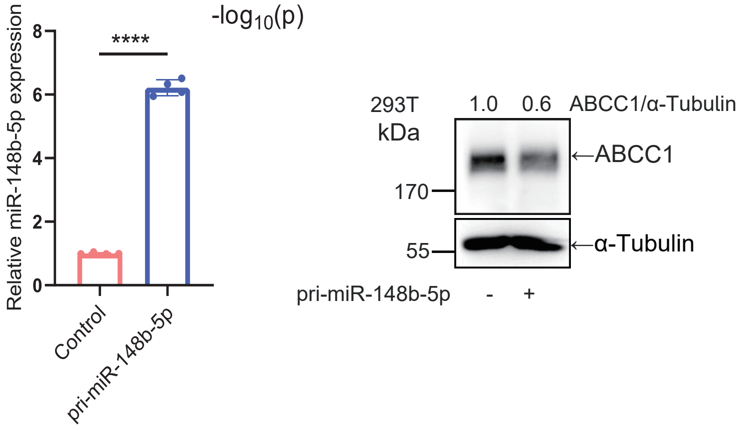

Figure S5. TRIM25-AGO2 axis regulates drug resistance via miR-148b-5p-mediated ABCC1 suppression. A Nuclear(N)-Cytosol(C) separation was performed in H1299 stable cell lines. Localization of AGO2 was detected with immunoblot. **B** Membrane(M)-Cytosol(C) separation was performed in H1299 stable cell lines. Localization of AGO2 was detected with immunoblot. **C** The list of the 25 miRNAs following the miR-148b-5p pattern. **D** Gene Ontology (GO) pathway enrichment analysis was performed on the predicted target genes of miR-148b-5p. **E** qPCR for endogenous miR-186-5p in A549 stable cell lines. Data were presented as mean ± SD, n = 3 independent samples. Statistical analysis was performed using two-tailed unpaired t-test, **** $p < 0.0001$. **F** Gene Ontology (GO) pathway enrichment analysis was performed using differentially expressed genes in mRNA-seq data. **G** KEGG pathway enrichment analysis for genes with 1.5-fold down-regulated (blue, upper panel) and up-regulated (orange, lower panel) mRNA transcripts when TRIM25 were knocked down and those that were rescued after AGO2 knockdown. **H** Western blotting analysis for endogenous ABCC1 in A549 stable cell lines. **I** Western blotting analysis for endogenous ABCC1 in 293T cells overexpressing pri-miR-148b-5p. For qPCR, data were presented a mean ± SD, n = 4. Statistical analysis was performed using two-tailed unpaired t-test, ****$p < 0.0001$. Band intensities were quantified by ImageJ software.

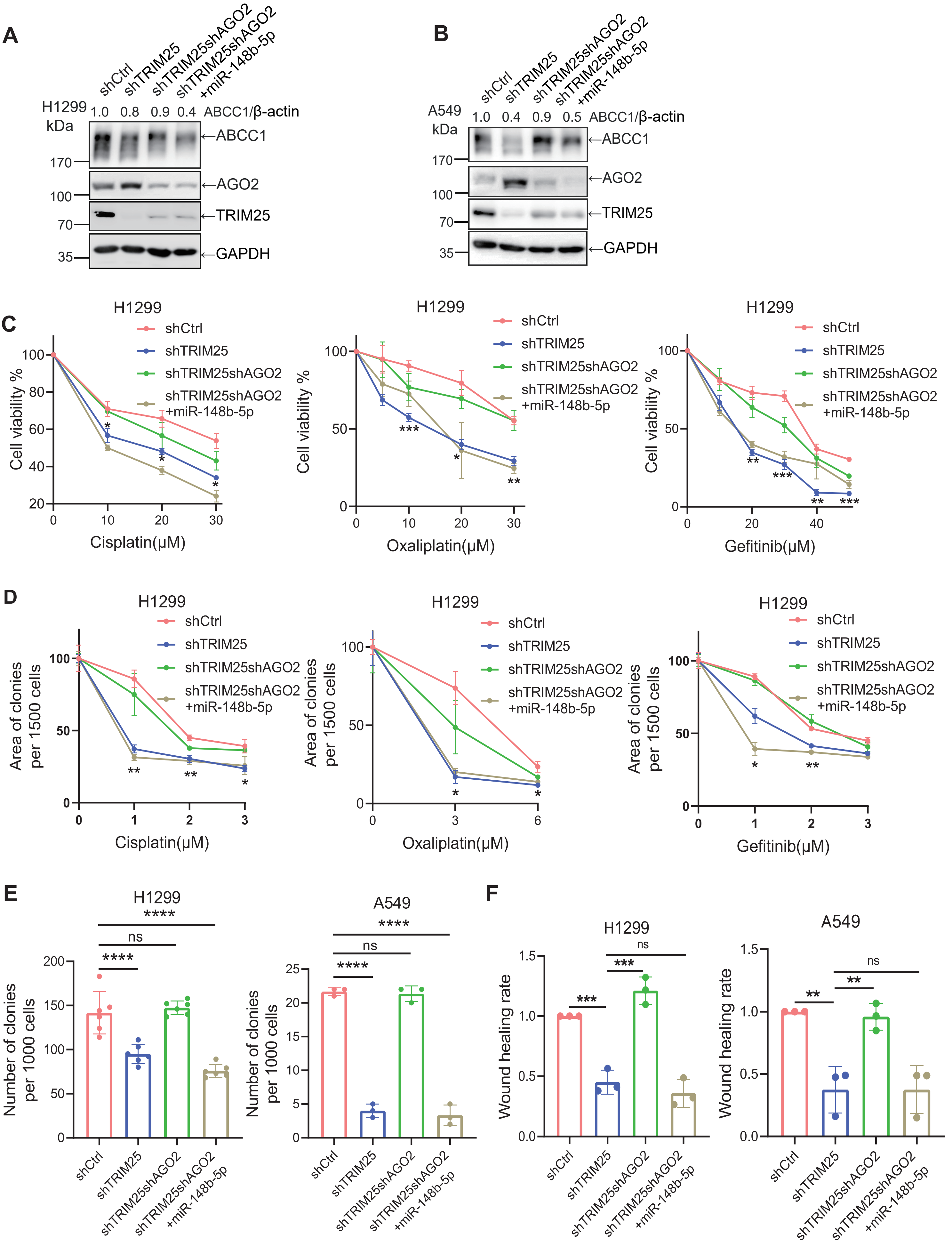

A
shCtrl
shTRIM25
shTRIM25shAGO2
shTRIM25shAGO2 +miR-148b-5p
H1299
1.0 0.8 0.9 0.4 ABCC1/β-actin
kDa
170
100
70
35
ABCC1
AGO2
TRIM25
GAPDH
B
A549
1.0 0.4 0.9 0.5 ABCC1/β-actin
C
H1299
Cell viability %
Cisplatin(μM)
Oxaliplatin(μM)
Gefitinib(μM)
D
Area of clonies per 1500 cells
E
Number of clonies per 1000 cells
F
Wound healing rate
ns

G

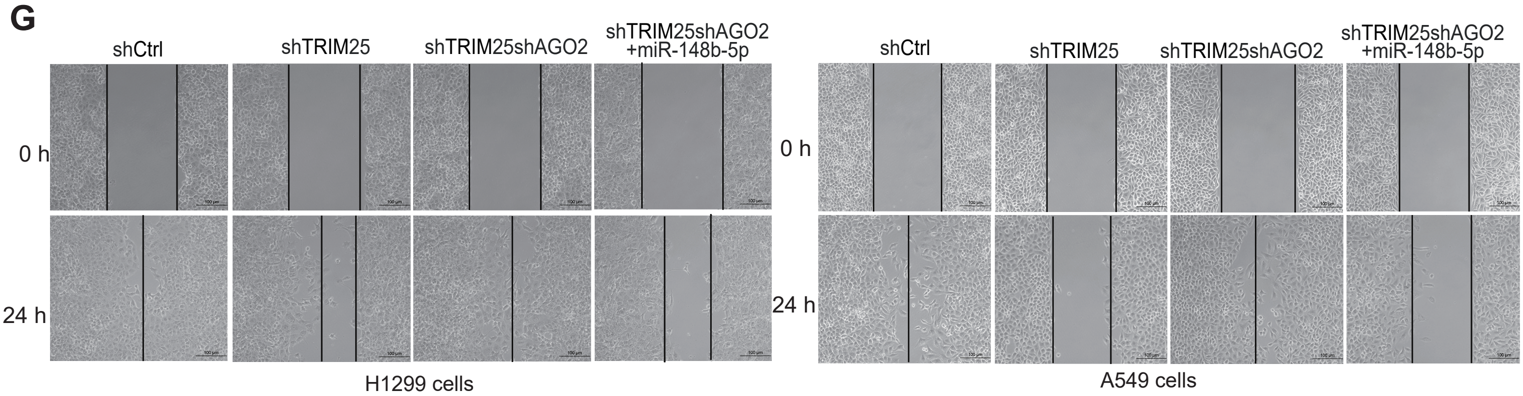

H

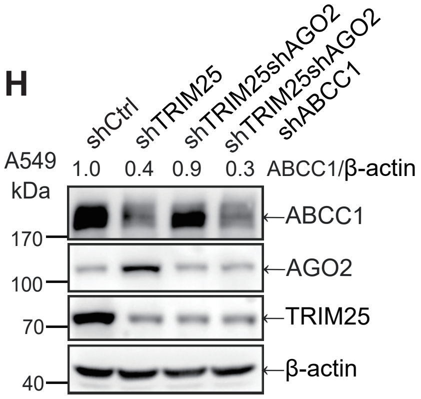

I

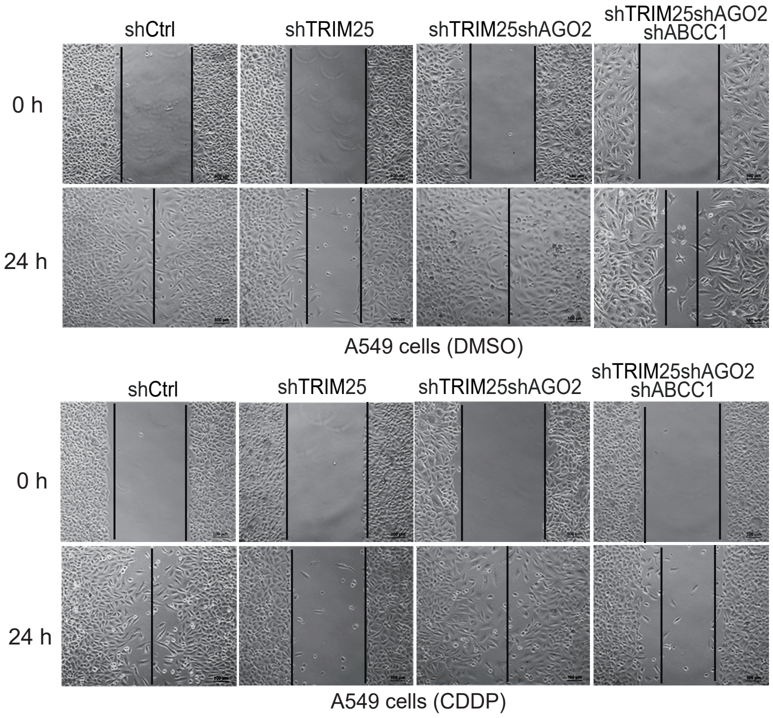

Figure S6. TRIM25-AGO2-miR-148b-5p axis governs ABCC1-mediated chemoresistance in cancer. A, B Construction of stable cell lines by overexpressing miR-148b-5p in H1299 **(A)** and A549 **(B)** cells. **C** CCK8 assay detecting the cell viability of H1299 stable cell lines under indicated Cisplatin, Oxaliplatin or Gefitinib concentration treatment. Data were presented as mean ± SD, n = 3. Statistical analysis was performed using two-way ANOVA. * $p < 0.05$, ** $p < 0.01$, ***$p < 0.001$. **D** Plate colony formation detecting clonogenic ability of H1299 stable cell lines under indicated Cisplatin, Oxaliplatin or Gefitinib concentration treatment. Data were presented as mean ± SD, n = 3. Statistical analysis was performed using two-way ANOVA. * $p < 0.05$, ** $p < 0.01$. **E** Statistics of colonies number in the soft-agar colony formation assay, H1299 and A549 cells were seeded at a density of 1000/well and cultured for 4 weeks. Data were presented as mean ± SD, n = 3 or 6. Statistical analysis was performed using two-tailed unpaired t-test. ****$p < 0.0001$. **F** The quantification results of wound healing assay in stable H1299 and A549 cells. Data were presented as mean ± SD, n = 3 independent experiments. Statistical analysis was performed using two-tailed unpaired t-test. ** $p < 0.01$,*** $p < 0$ .001. **G** Wound healing assay, photographs were taken at 0 h and 24 h later in H1299 and A549 cells, scale: 100 μm. **H** Construction of stable cell lines by knocking down ABCC1 in A549 cells. **I** Wound healing assay, photographs were taken at 0 h and 24 h later in A549 cells, scale: 100 μm.

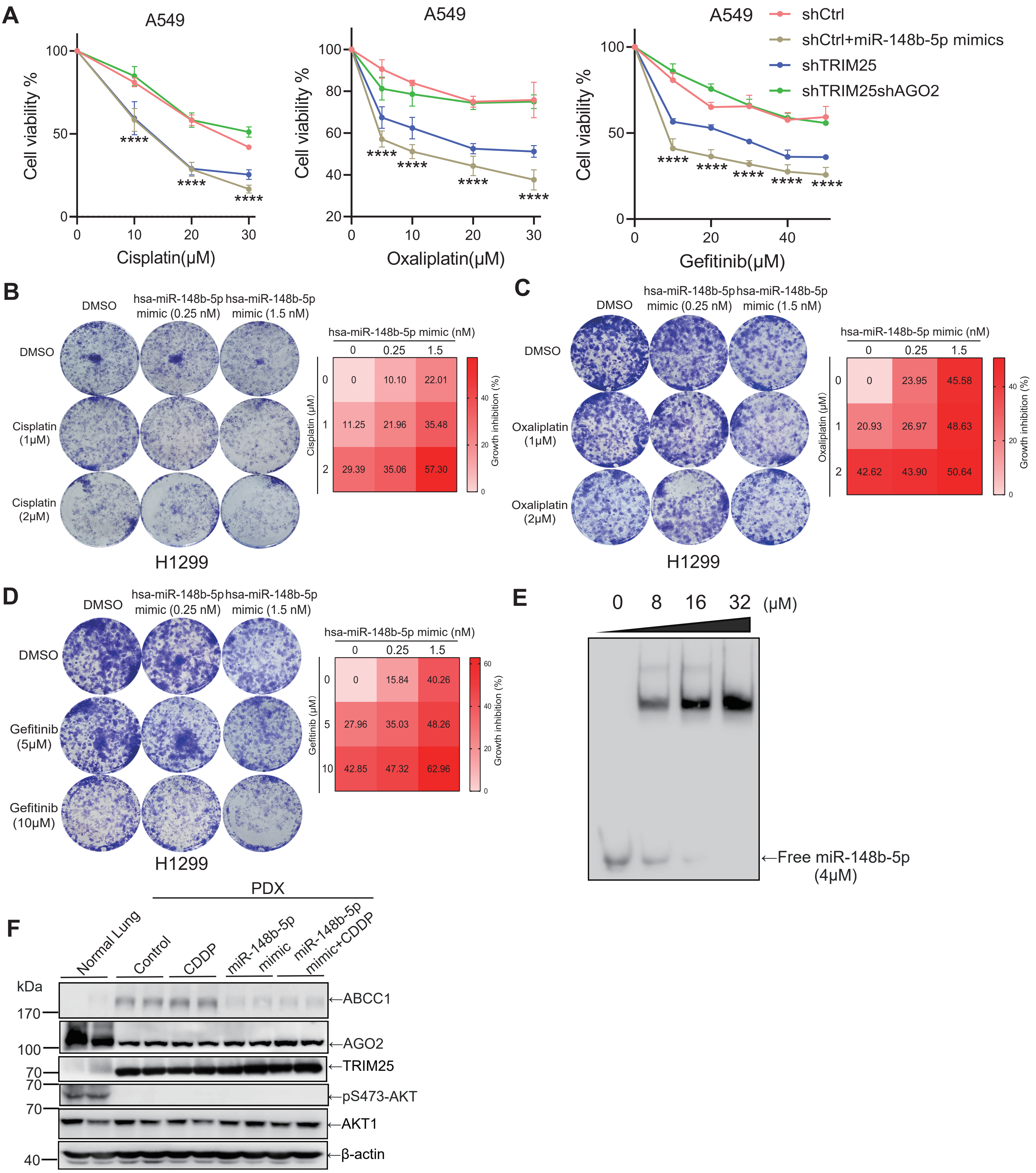

Figure S7. The combination of miR-148b-5p and CDDP enhanced the anti-tumor effect in NSCLC PDX model. **A** A549 shCtrl cells were transfected with miR-148b-5p mimics for 24 hours before plating. Cell viability was measured by CCK-8 assay following treatment with indicated concentrations of Cisplatin, Oxaliplatin, or Gefitinib across four groups. Data were presented as mean ± SD, n = 3. Statistical analysis was performed using two-way ANOVA. ****$p < 0.0001$. **B-D** H1299 cells were treated with Cisplatin **(B)**, Oxaliplatin **(C)** or Gefitinib **(D)** and miR-148b-5p mimics or their combination at the indicated concentrations. Cells were fixed and stained after 10-12 days. Representative data from three independent experiments. **E** EMSA experiment to detect the direct interaction between CABRi and miR-148b-5p mimic. **F** Western blot analysis of ABCC1, AGO2, TRIM25, pS473-AKT, and total AKT1 in normal human lung tissue and PDX tumors from the indicated treatment groups.
