# Supplementary material for: Targeting the TRIM25–AGO2–miR-148b-5p–ABCC1 axis overcomes chemoresistance in non-small cell lung cancer
Source: Cell Death Dis. 2026 Apr 30;17(1):579. doi: 10.1038/s41419-026-08802-1 (PMC13276176; doi:10.1038/s41419-026-08802-1)
Supplement: Supplementary file 2 — Supplementary Table S1-S2 [file 41419_2026_8802_MOESM2_ESM.docx]

**Table S1. Reagents and Antibodies**

| Reagents | Source |
| --- | --- |
| MG132 | Sangon |
| Cycloheximide | Sigma-Aldrich |
| IGF-1 | Sangon |
| Insulin | YEASEN |
| EGF | Proteintech |
| Cisplatin | Selleckchem |
| Gefitinib | Selleckchem |
| Oxaliplatin | Hengrui Medicine |
| Lipofectamine 2000 | Invitrogen |
| TRIzol Reagent | Sigma-Aldrich |
| Puromycin | YEASEN |
| Cell counting kit (CCK8) | YEASEN |

| Antibodies | Source |
| --- | --- |
| anti-AGO2 | Cell Signaling Technology |
| anti-TRIM25 | Proteintech |
| anti-ABCC1 | HUABIO |
| Phospho-(Ser/Thr) Akt Substrate Antibody | Cell Signaling Technology |
| anti- Phospho-Akt (Ser473) | Cell Signaling Technology |
| anti- Akt | Cell Signaling Technology |
| anti-α-tubulin | Proteintech |
| anti-β-actin | Proteintech |
| anti-GAPDH | Proteintech |
| anti-Ubiquitin | Proteintech |
| anti-GFP | Proteintech |
| anti-His | Proteintech |
| anti-GST | Proteintech |
| anti-HA | Covance |
| anti-Myc | Cell Signaling Technology |
| anti-Flag | Sigma-Aldrich |
| normal mouse IgG | Santa Cruz Biotechnology |
| normal rabbit IgG | Santa Cruz Biotechnology |

**Table S2. Sequences used for plasmid construction primers, shRNAs, qRT-PCR primers, Northern blot and EMSA**

**Primers for plasmid construction**

| Primer name | Sequence (5' to 3') |
| --- | --- |
| TRIM25 C50/53S-FWD | CTGTCCCCGCAGTCCCGCGCCGTCTAC |
| TRIM25 C50/53S-REV | GGGACTGCGGGGACAGGTATGGCGAGC |
| TRIM25 ΔRING- FWD | GGGTACCATGTCGAGAGCACTGGATGATGTGAGAA |
| TRIM25 ΔRING-REV | TTGCGGCCGCCTACTTGGGGGAGCAGATG |
| TRIM25 ΔCoiled-coil- FWD | CTGGAGAAAGCATCAAAACTGCG |
| TRIM25 ΔCoiled-coil- REV | CGCCCCGTTGATCTGACTGT |
| TRIM25 ΔPRYSPRY- FWD | GGGTACCATGGCAGAGCTGTGCCCC |
| TRIM25 ΔPRYSPRY-REV | TTGCGGCCGCCTAAAACTCGAACTCATCCCTCTT |
| TRIM25 S158A-FWD | CGCAAATGTGCCCAGCACAATCGG |
| TRIM25 S158A -REV | GCTGGGCACATTTGCGGCGCAA |
| TRIM25 T319A -FWD | GAATCTCAGCAAAGCCAGTCTACA |
| TRIM25 T319A -REV | GCTTTGCTGAGATTCCTCGCAGT |
| pGEX-4T-1-TRIM25-FWD | GGAATTCATGGCAGAGCTGTGCCCCCT |
| pGEX-4T-1- TRIM25-REV | ACGCGTCGACCTACTTGGGGGAGCAGATGGAGAG |
| pri-miR-148b-5p-FWD | GGAATTCGGAGGTTGTAGTGAGCGGAGTT |
| pri-miR-148b-5p-REV | TTGCGGCCGCAGGGCTATGCCATAAAACTAGCC |
| ABCC1 3’UTR-FWD | CCTCGAGGCCCCAGAGCTGGCATATCT |
| ABCC1 3’UTR-  REV | TTGCGGCCGCACTGCAAATGTTCGCATTTCCAG |
| ABCC1 3’UTR Mut-FWD | TTGAGCAGGGCCTATATGCCAG |
| ABCC1 3’UTR Mut- REV | GACACCAGATATGCCAGCTCTGG |

**shRNA sequences**

| Primer name | Sequence (5' to 3') |
| --- | --- |
| pLKO.1-shTRIM25-1 FWD | CCGGCCGGAACAGTTAGTGGATTTACTCGAGTAAATCCACTAACTGTTCCGGTTTTTG |
| pLKO.1- shTRIM25-1 REV | AATTCAAAAACCGGAACAGTTAGTGGATTTACTCGAGTAAATCCACTAACTGTTCCGG |
| pLKO.1-shTRIM25-2 FWD | CCGGGAACTGAACCACAAGCTGATACTCGAGTATCAGCTTGTGGTTCAGTTCTTTTTG |
| pLKO.1- shTRIM25-2 REV | AATTCAAAAAGAACTGAACCACAAGCTGATACTCGAGTATCAGCTTGTGGTTCAGTTC |
| pLKO.1-shTRIM25-3 FWD | CCGGGTGCCCGATTCCTCTTAGAGACTCGAGTCTCTAAGAGGAATCGGGCACTTTTTG |
| pLKO.1- shTRIM25-3 REV | AATTCAAAAAGTGCCCGATTCCTCTTAGAGACTCGAGTCTCTAAGAGGAATCGGGCAC |
| pLKO.1-shAGO2 FWD | CCGGTATCGAACATGAGACGTCATTGCTCGAGCA  ATGACGTCTCATGTTCGATTTTTTTG |
| pLKO.1- shAGO2REV | AATTCAAAAAAATCGAACATGAGACGTCATTGCT  CGAGCAATGACGTCTCATGTTCGATA |
| shABCC1 plasmid | pLV3-U6-ABCC1-shRNA1 (CCTGGGCTTATTTCGGATCAA)-CopGFP-Puro (Purchased from Miaoling Bio) |

**Primers for qRT-PCR quantification**

| Primer name | Sequence (5' to 3') |
| --- | --- |
| RT-miR-148b-5p | GTCGTATCCAGTGCAGGGTCCGAGGTATTCGCACTGGATACGACGCCTGA |
| qPCR-miR-148b-5p-FWD | GCCTGAAGTTCTGTTATACA |
| qPCR-miRNA-REV | GTGCAGGGTCCGAGGT |
| U6-FWD | CGCTTCGGCAGCACATATAC |
| U6-REV | AGGGGCCATGCTAATCTTCT |
| β-actin-qPCR-FWD | GCACAGAGCCTCGCCTT |
| β-actin-qPCR-REV | GTTGTCGACGACGAGCG |

**Probe sequences for Northern blot and EMSA**

| Name | Sequence (5' to 3') |
| --- | --- |
| miR-148b-5p- Northern probe | GCCTGAGTGTATAACAGAACTT |
| U6- Northern probe | TGTGCTGCCGAAGCGAGCAC |
| miR-148b-5p-mimic-biotin-sense | GCCUGAGUGUAUAACAGAACUU-biotin |
| miR-148b-5p-mimic-antisense | AAGUUCUGUUAUACACUCAGGC |
