# Supplementary material for: Targeting the TRIM25–AGO2–miR-148b-5p–ABCC1 axis overcomes chemoresistance in non-small cell lung cancer
Source: Cell Death Dis. 2026 Apr 30;17(1):579. doi: 10.1038/s41419-026-08802-1 (PMC13276176; doi:10.1038/s41419-026-08802-1)

Fig.1 original images

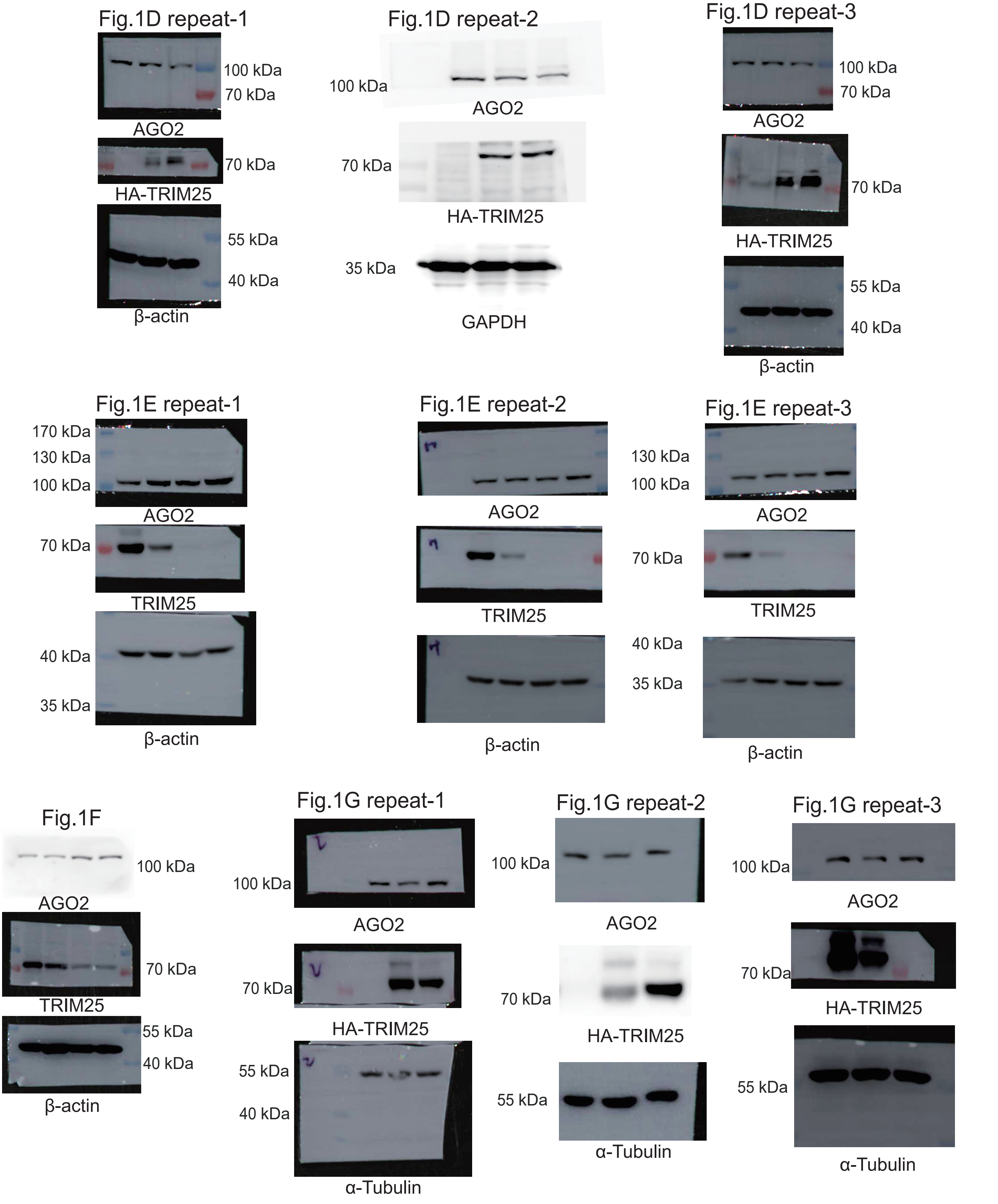

Fig.1 original images

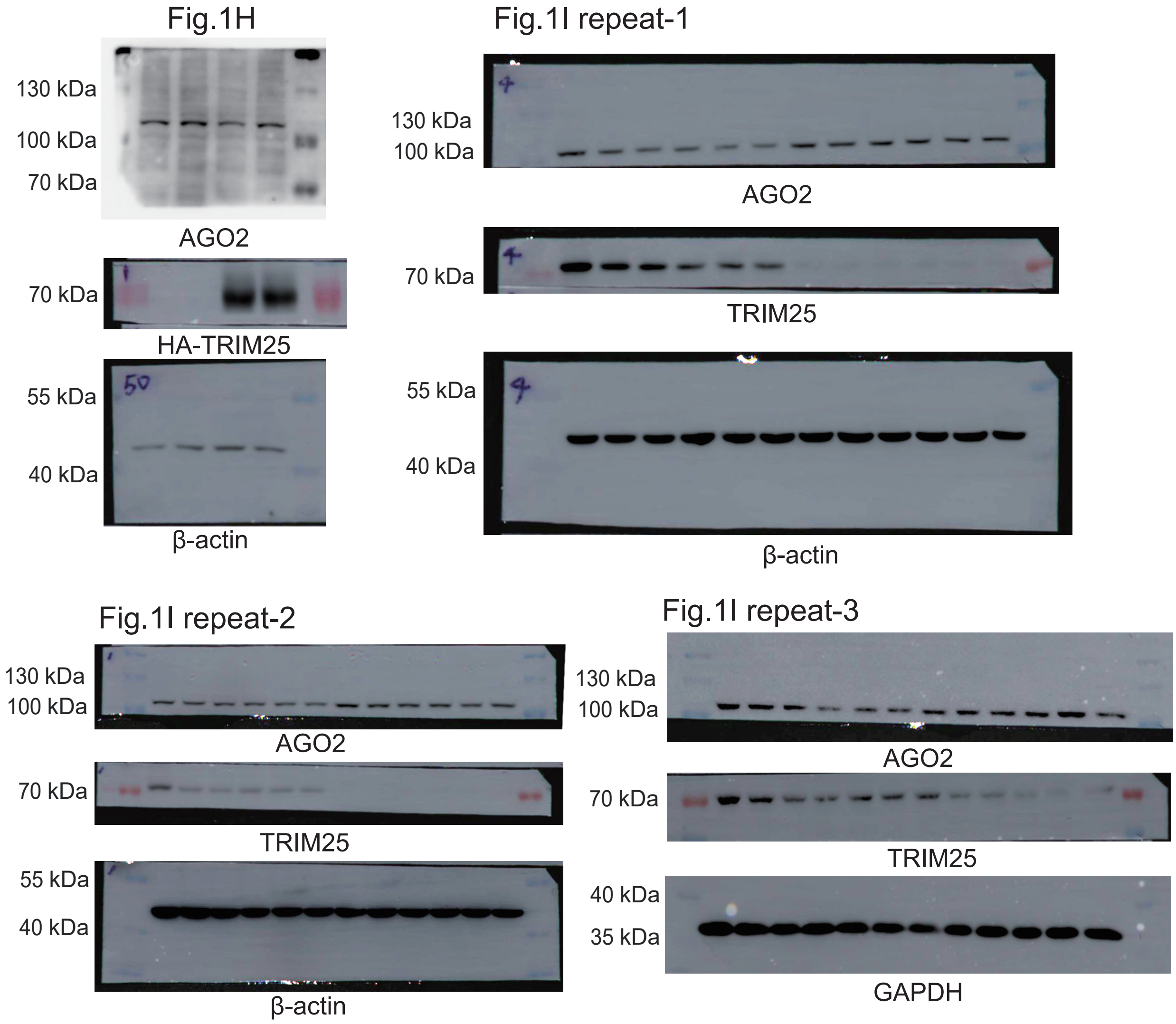

Fig.2 original images

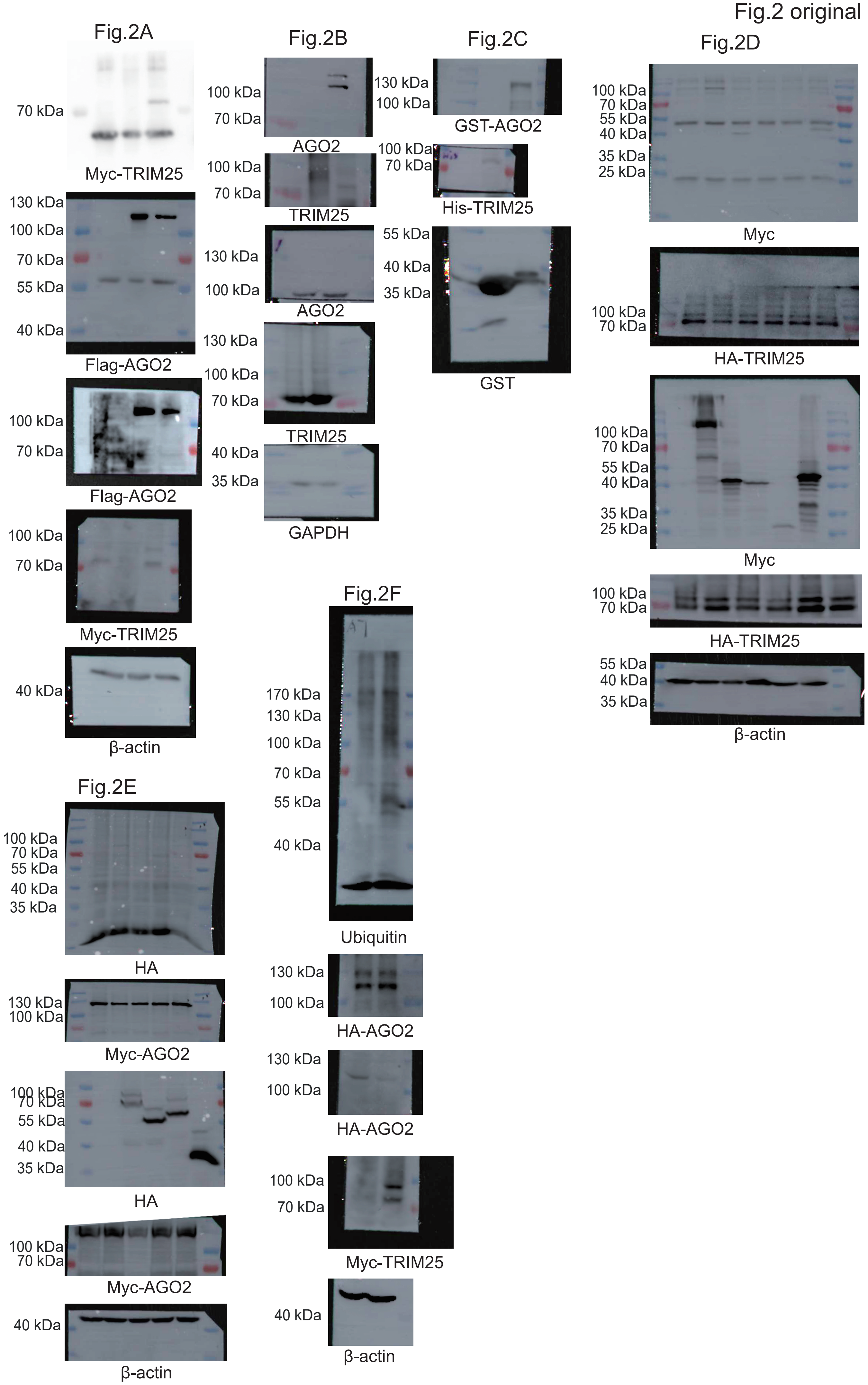

Fig.2 original images

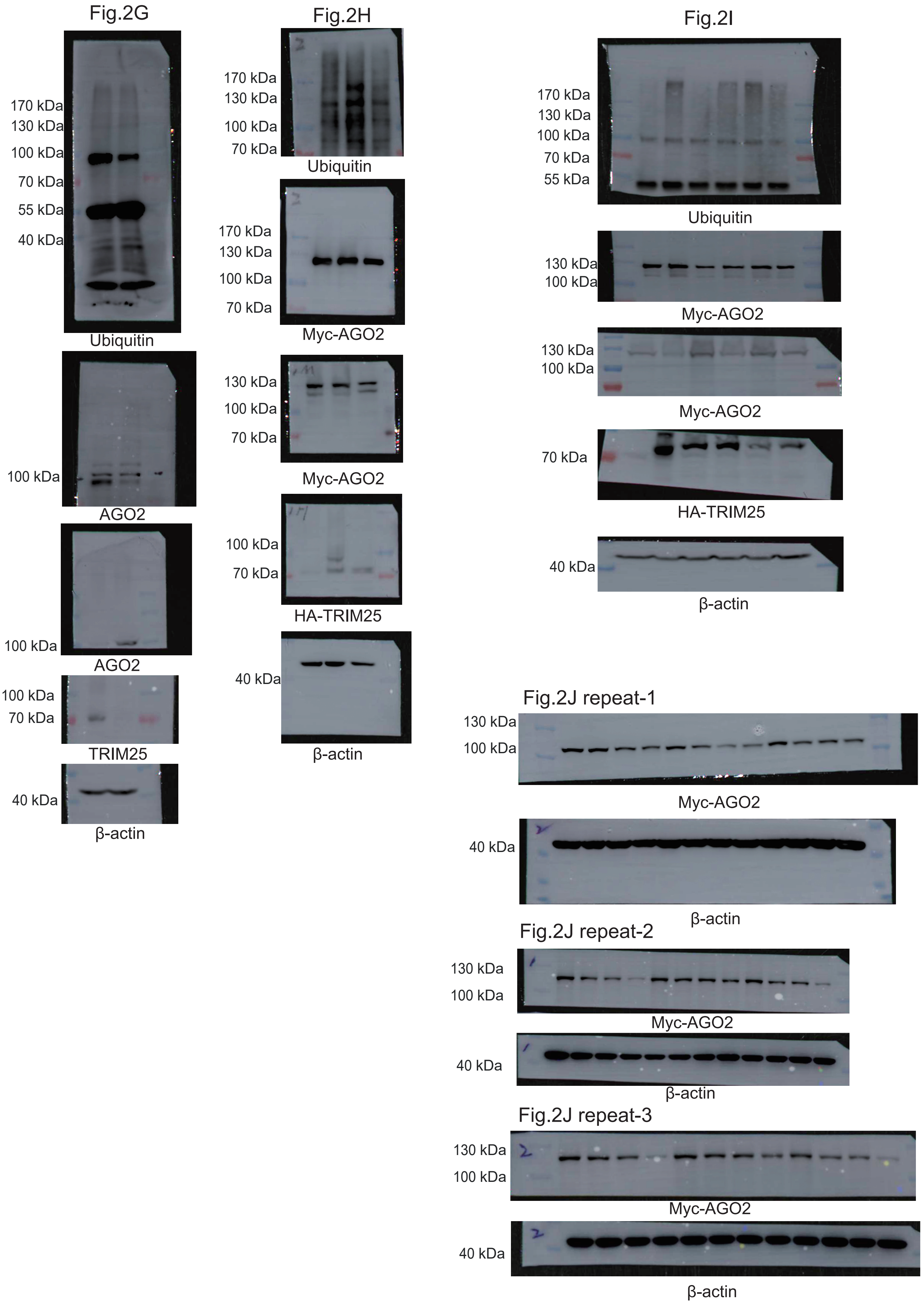

Fig.3 original images

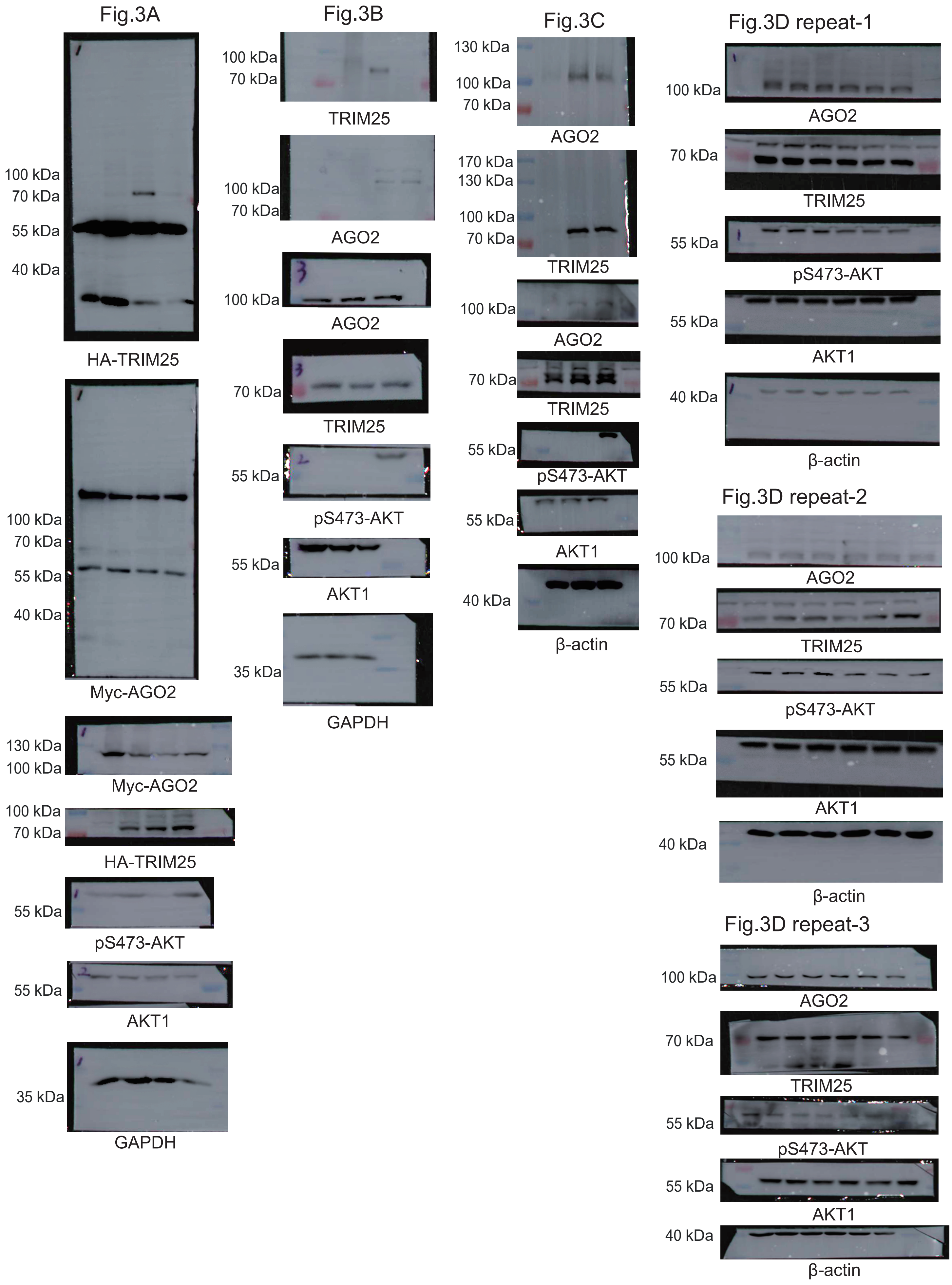

Fig.3 original images

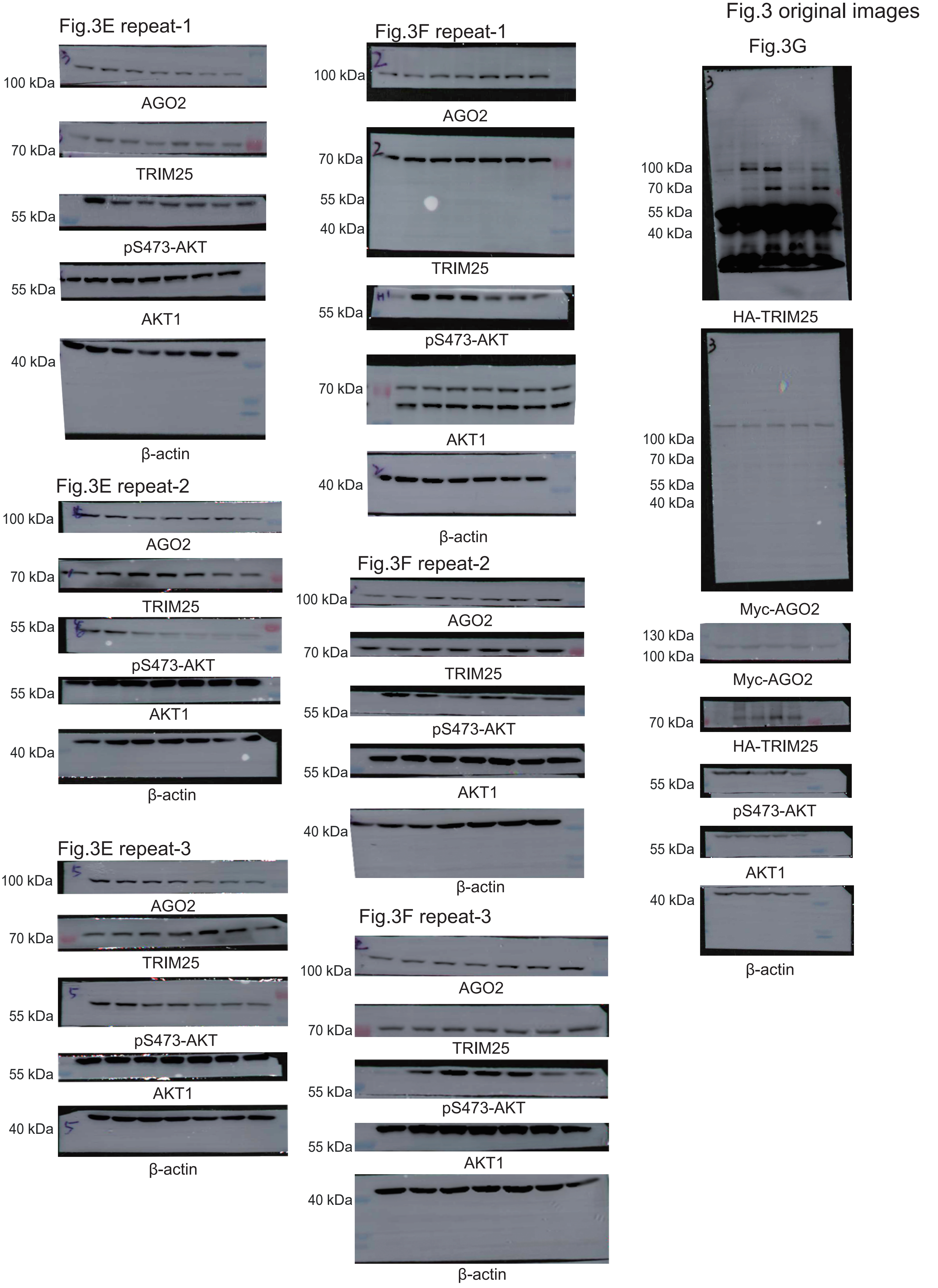

Fig.3 original images

Fig.3H

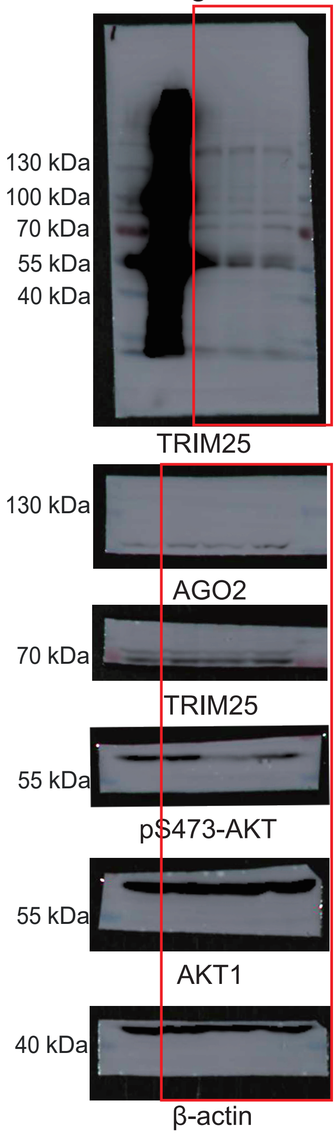

Fig.3I

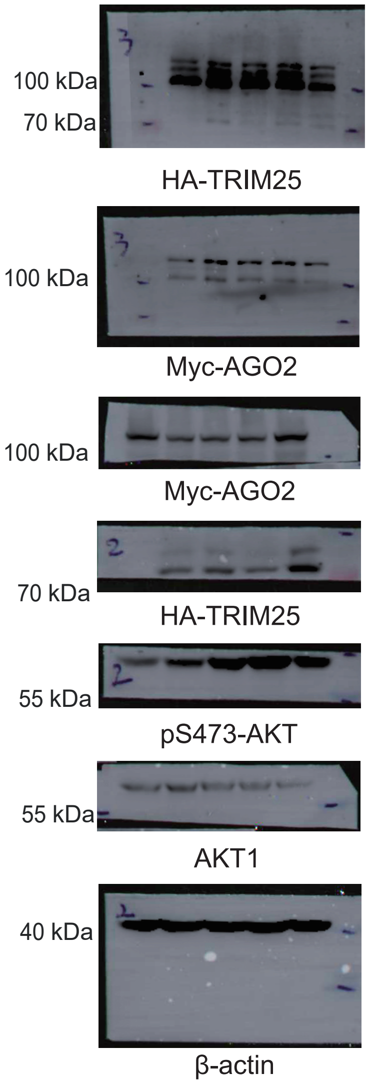

Fig.3J

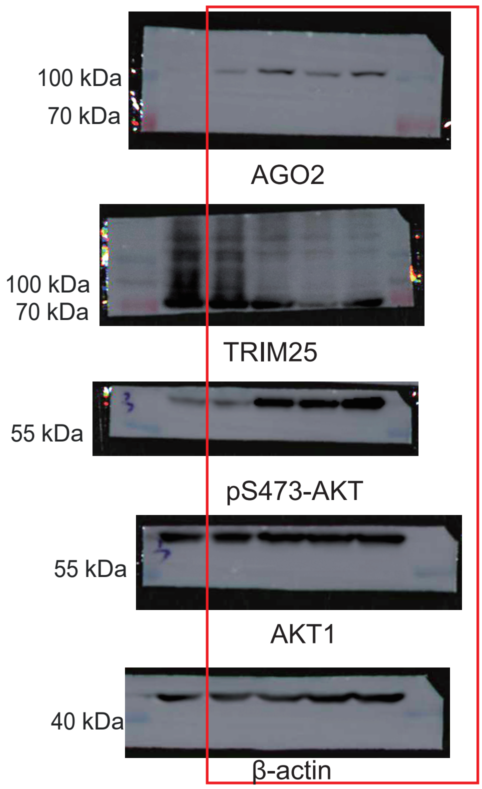

Fig. 4 original images

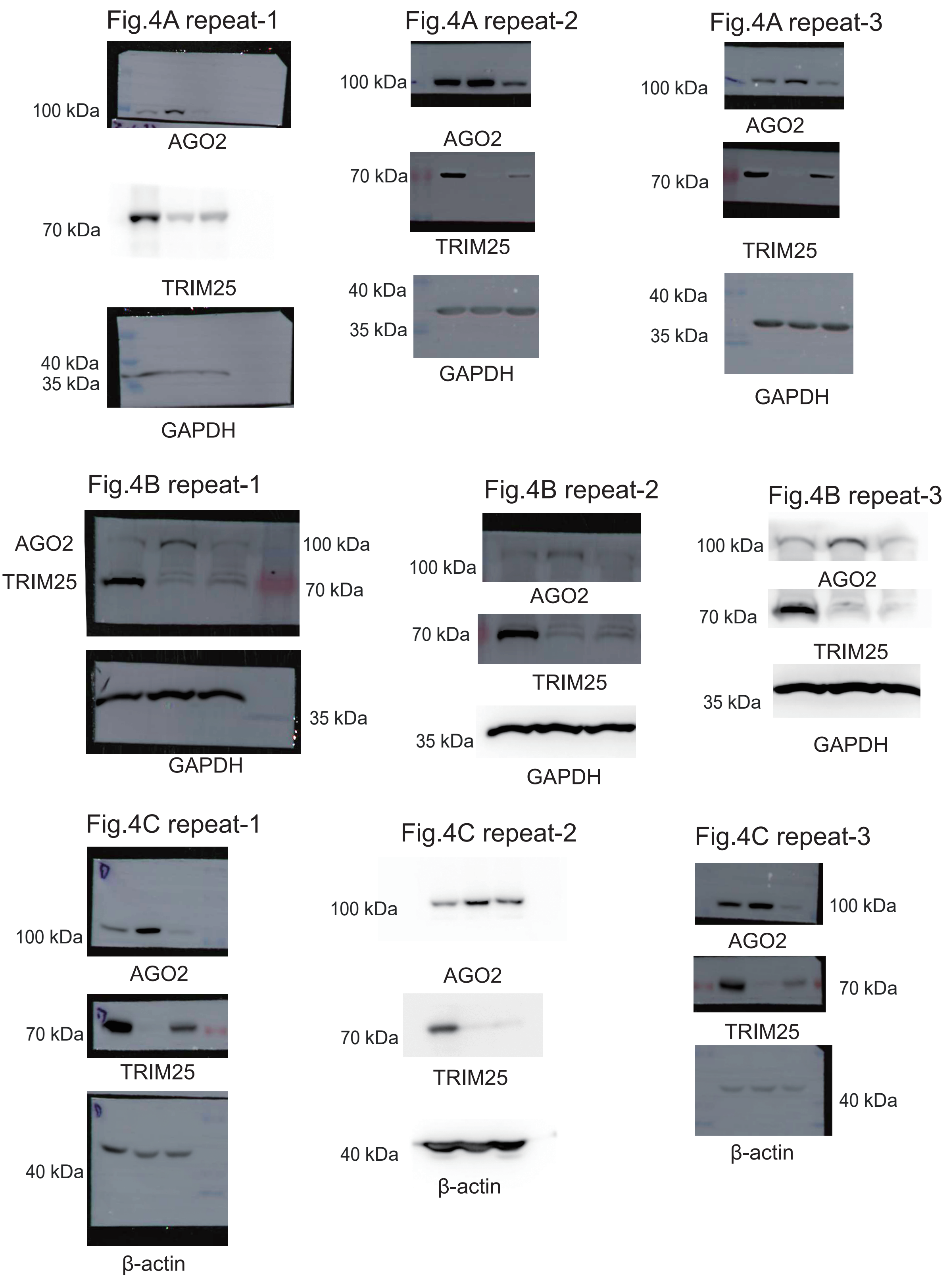

Fig. 5 original images

Fig.5A

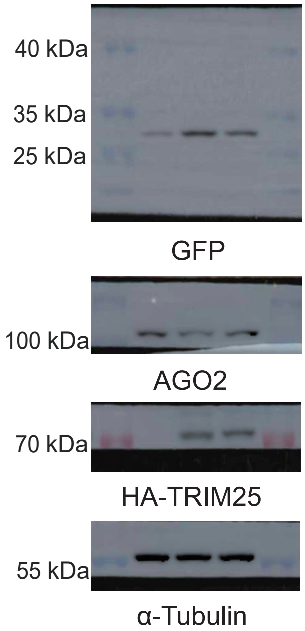

Fig.5B

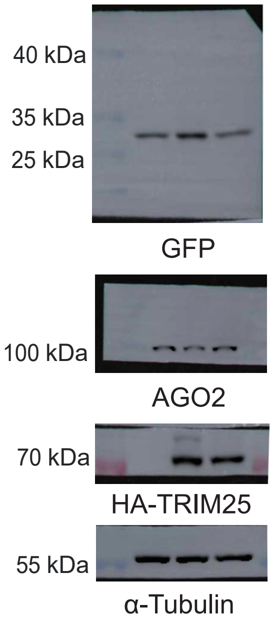

Fig.5D

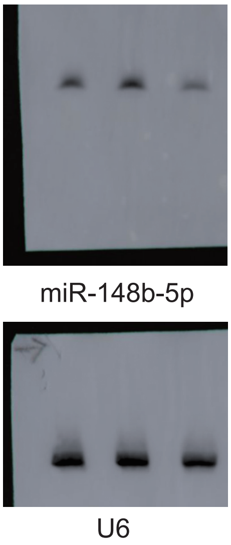

Fig.5H

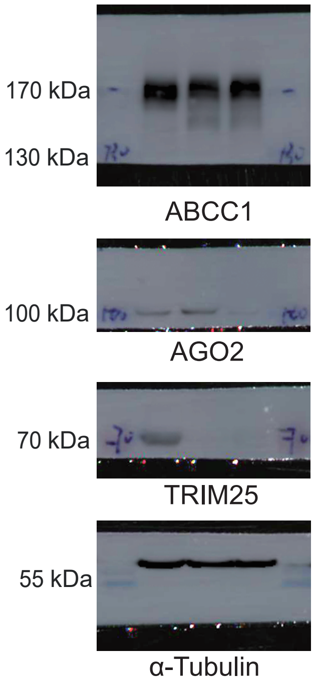

Fig.5K

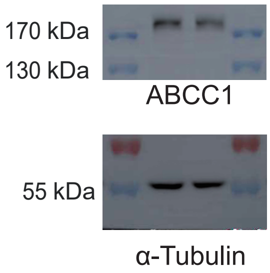

Fig.6 original images

Fig.6C

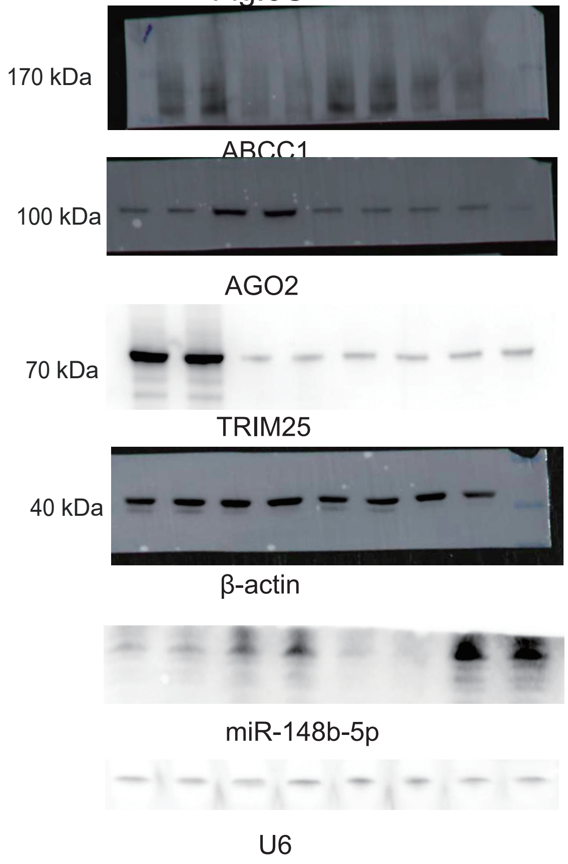

Fig.6G

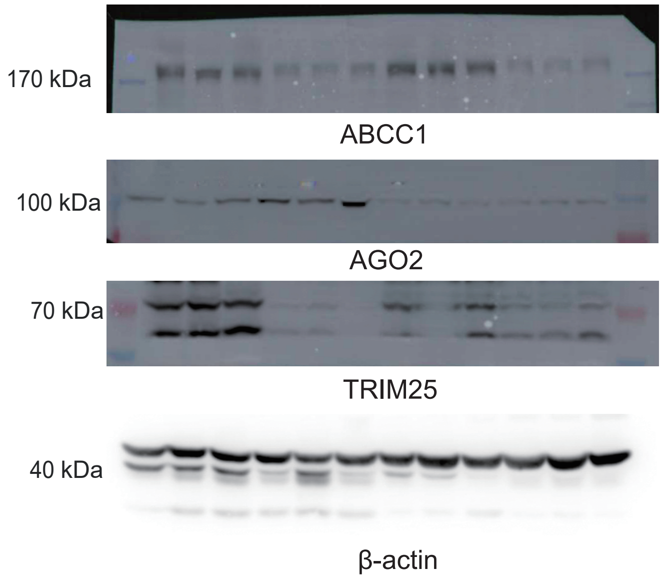

Fig.7H

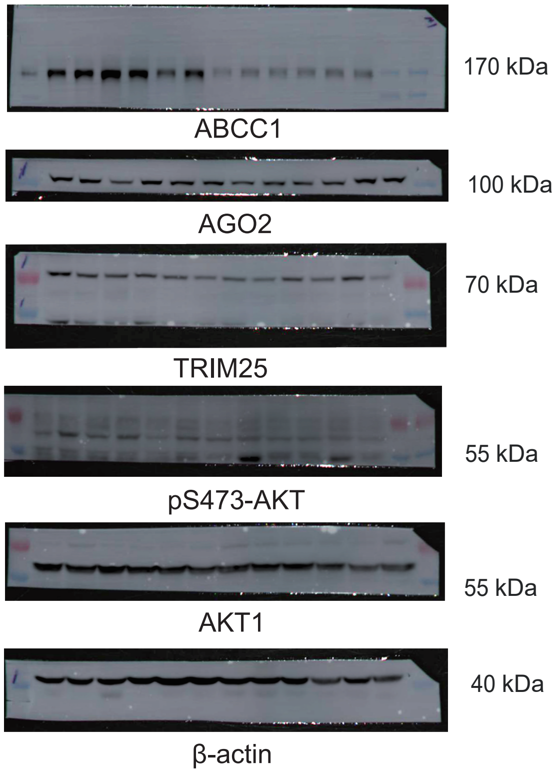
170 kDa
ABCC1
100 kDa
AGO2
70 kDa
TRIM25
55 kDa
pS473-AKT
55 kDa
AKT1
40 kDa
β-actin

Fig.S1 original images

Fig.S1E repeat-1

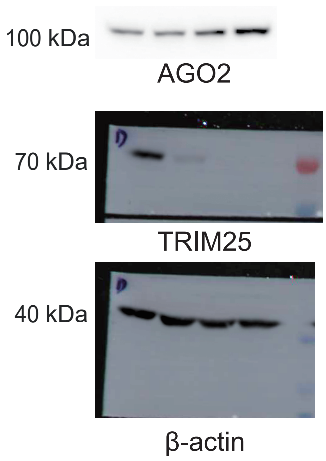

Fig.S1E repeat-2

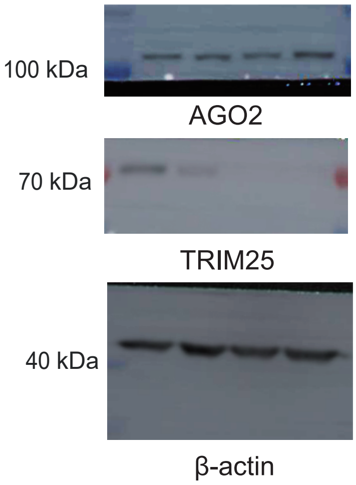

Fig.S1E repeat-3

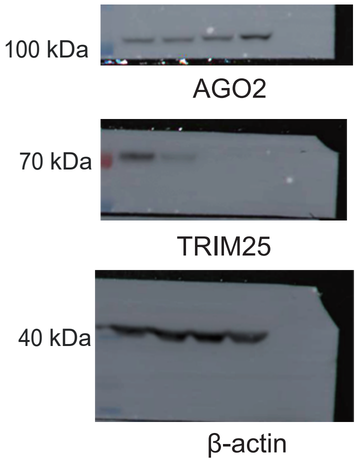

Fig.S1F

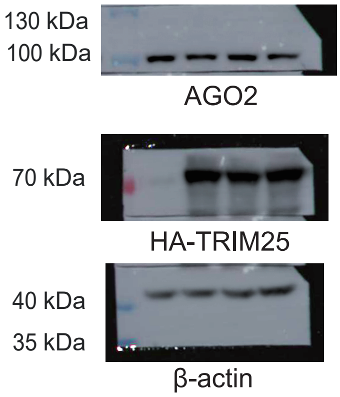

Fig.S1G repeat-1

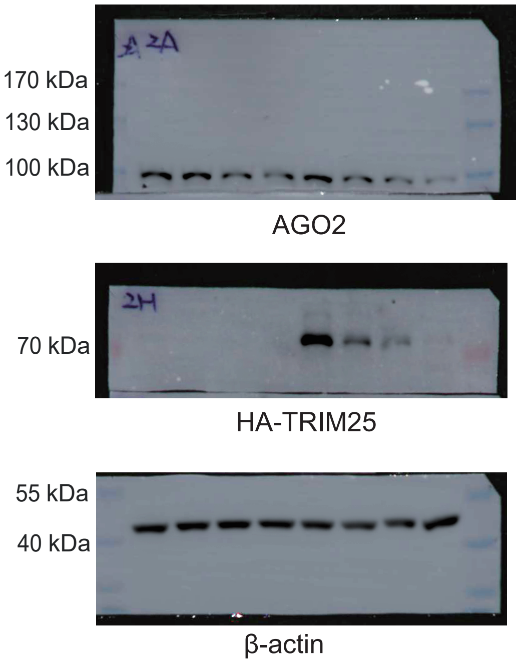

Fig.S1G repeat-2

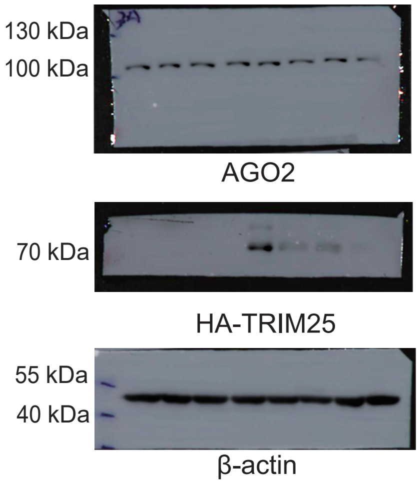

Fig.S1G repeat-3

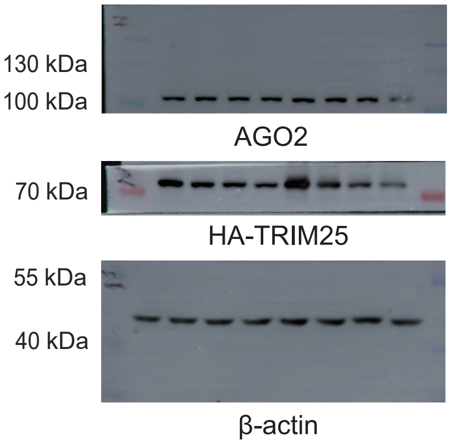

Fig.S2 original images

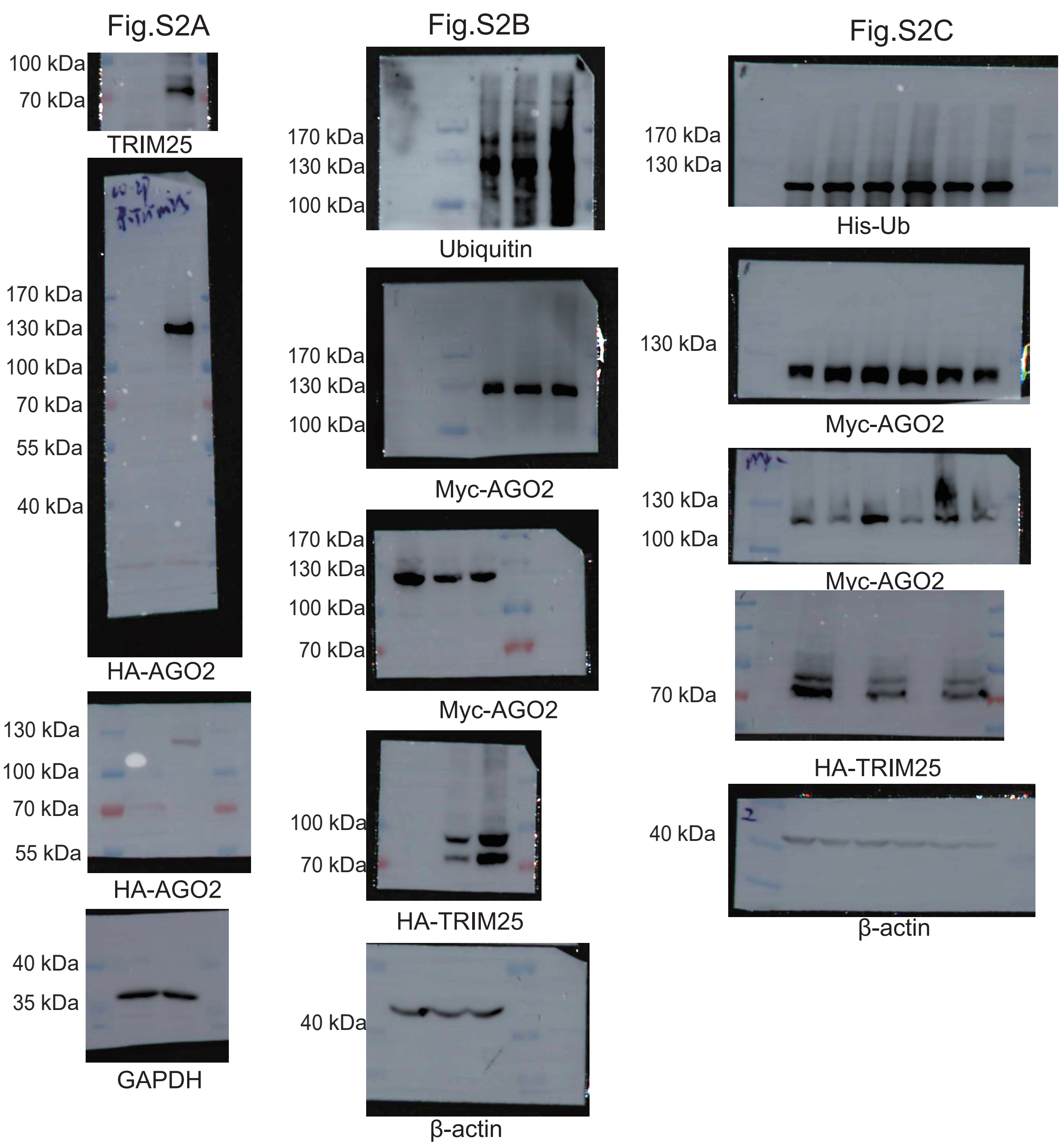

Fig.S3 original images

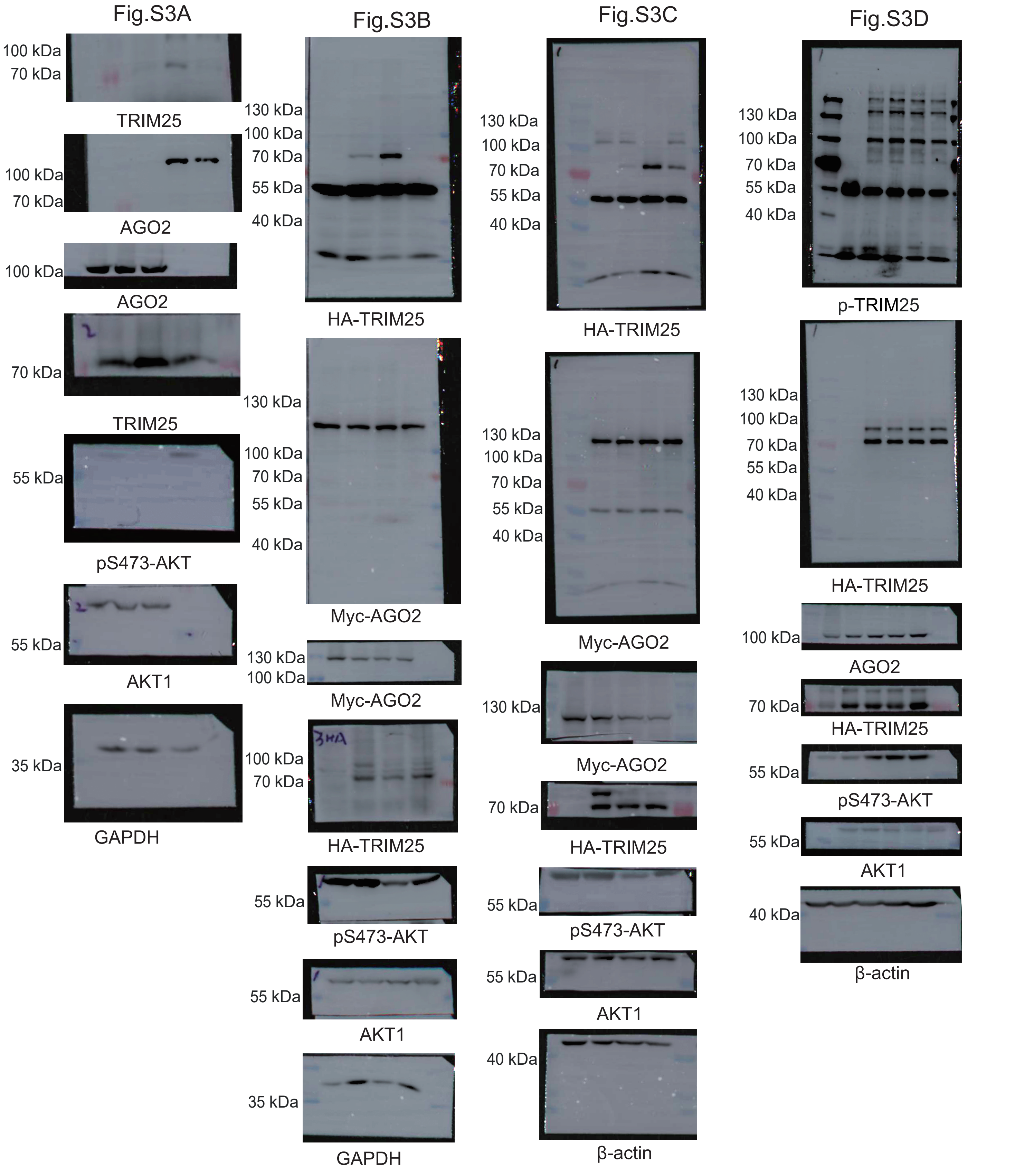

Fig.S5 original images

Fig.S5A repeat-1

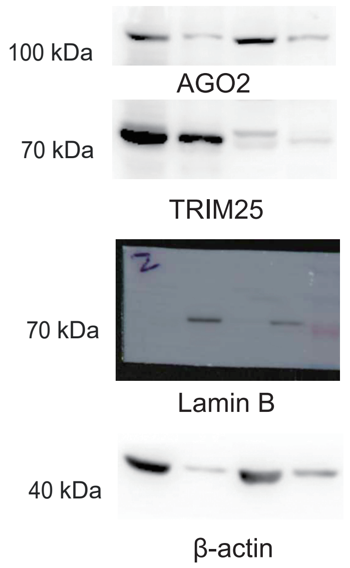

Fig.S5A repeat-2

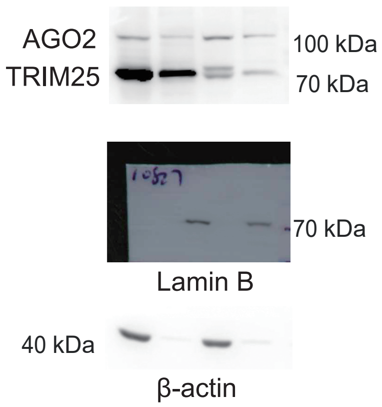

Fig.S5A repeat-3

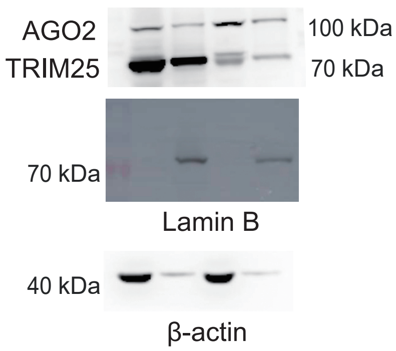

Fig.S5B

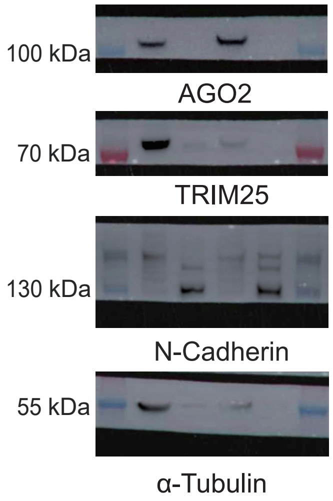

Fig.S5H

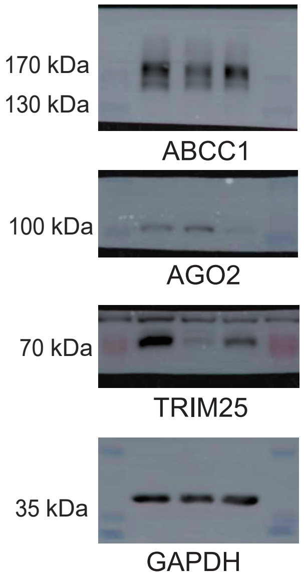

Fig.S5I

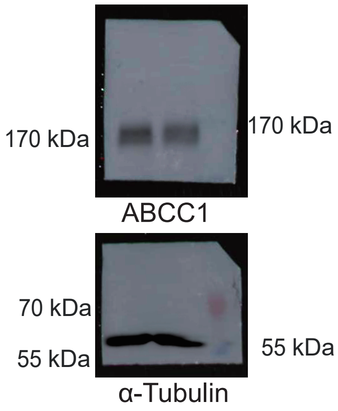

Fig.S6 original images

Fig.S6A

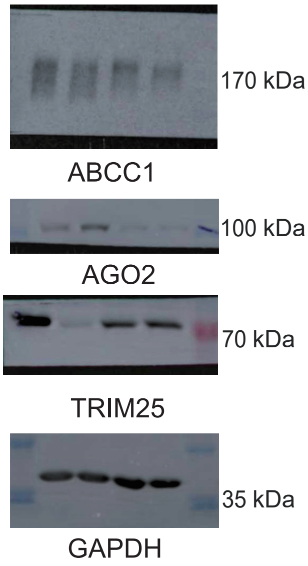

Fig.S6B

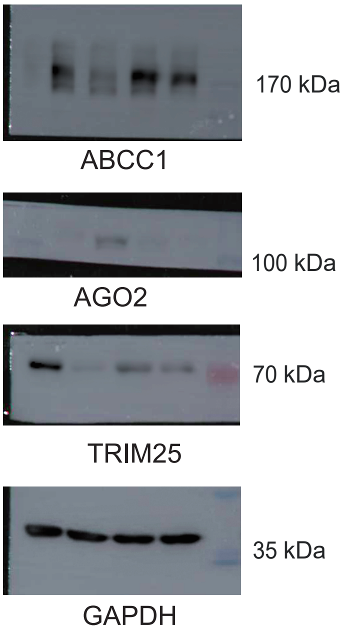

Fig.S6H

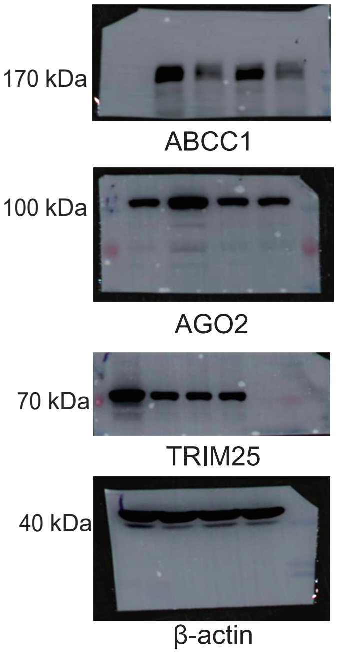

Fig.S7 original images

Fig.S7E

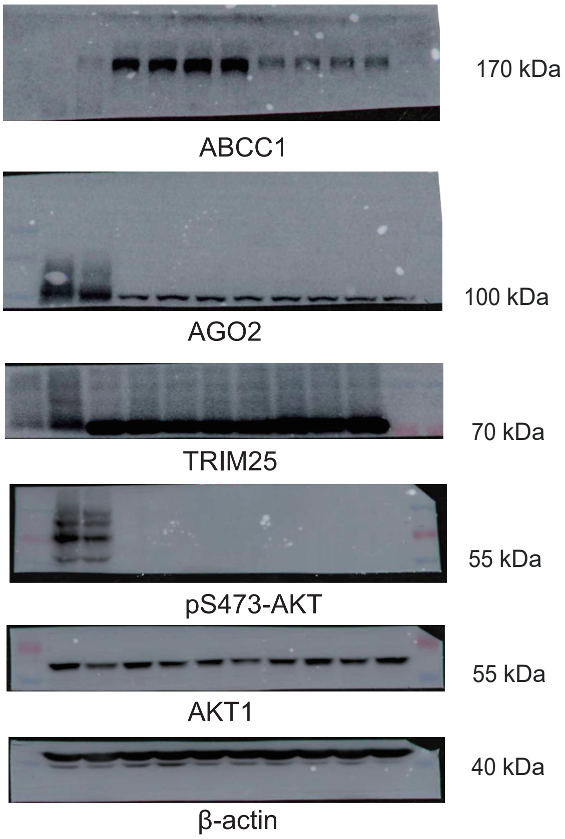

Supplement: Supplementary file 4 — Uncropped original western blots [file 41419_2026_8802_MOESM4_ESM.pdf]
